# The Fate of Copper Added to Surface Water: Field, Laboratory, and Modeling Studies

**DOI:** 10.1002/etc.4440

**Published:** 2019-06-24

**Authors:** Kevin J. Rader, Richard F. Carbonaro, Eric D. van Hullebusch, Stijn Baken, Katrien Delbeke

**Affiliations:** ^1^ Mutch Associates Ramsey New Jersey USA; ^2^ Chemical Engineering Department Manhattan College, Riverdale New York USA; ^3^ Institut de Physique du Globe de Paris, Sorbonne Paris Cité, Université Paris Diderot Paris France; ^4^ European Copper Institute Brussels Belgium

**Keywords:** Metals, Metal speciation, Environmental fate, Fate modeling, Copper, Hazard assessment

## Abstract

The fate and effects of copper in the environment are governed by a complex set of environmental processes that include binding to inorganic and organic ligands in water, soil, and sediments. In natural waters, these interactions can limit copper bioavailability and result in copper transport from the water column to the sediment. In the present study, data on the fate of copper added to lakes, microcosms, and mesocosms were compiled and analyzed to determine copper removal rates from the water column. Studies on copper behavior in sediment were also reviewed to assess the potential for remobilization. A previously developed, screening‐level fate and transport model (tableau input coupled kinetic equilibrium transport–unit world model [TICKET–UWM]) was parameterized and applied to quantify copper removal rates and remobilization in a standardized lake setting. Field and modeling results were reconciled within a framework that links copper removal rates to lake depths and solids fluxes. The results of these analyses provide converging evidence that, on a large scale, copper is removed relatively quickly from natural waters. For the majority of studies examined, more than 70% of the added copper was removed from the water column within 16 d of dosing. This information may be useful in the context of environmental hazard and risk assessment of copper. *Environ Toxicol Chem* 2019;38:1386‒1399. © 2019 The Authors. *Environmental Toxicology and Chemistry* published by Wiley Periodicals, Inc. on behalf of SETAC.

## INTRODUCTION

The fate of copper in surface waters is governed by many complex environmental processes. Copper in surface waters occurs predominantly as the cupric form, Cu(II). When dissolved copper is introduced into the environment, the cupric ion typically binds to inorganic and organic ligands contained within water, soil, and sediments. In natural waters, copper ions form stable complexes with ‐NH_2_, ‐SH, and, ‐OH groups of dissolved organic matter. For example, in typical natural waters, more than 98% of the dissolved copper may be complexed to dissolved natural organic matter (e.g., fulvic acids; Tipping 1994; Bryan et al. 2002). In sediment, copper may bind to various inorganic and organic constituents, such as hydrous manganese oxides and iron oxides, clay, organic matter, and sulfides. The distribution of copper among these forms depends on pH, the oxidation‐reduction potential in the local environment, and the presence of competing metal ions and inorganic anions. The strong tendency of copper to bind to various dissolved ligands and solid phases has important implications for its fate, because such binding will affect the transformations and fluxes of copper between various compartments in the aquatic environment.

Information on environmental fate is relevant for assessing whether chemicals constitute environmental hazards. A hazard is an inherent or intrinsic property of a substance that has the potential to cause adverse effects in a living organism (Organisation for Economic Co‐operation and Development 2003). The Globally Harmonized System for Classification and Labelling of Chemicals (GHS) contains uniform rules to assess the hazard of chemicals (United Nations 2017). The GHS assessment of environmental hazard considers degradability, recognizing that, in the event of a spillage or accident, effects of rapidly degraded substances are localized and of short duration. However, the GHS (section A9.7.1.5) recognizes that the unique aspects of metal chemistry complicate the assessment:

"For inorganic compounds and metals, clearly the concept of degradability, as it has been considered and used for organic substances, has limited or no meaning. Rather, the substance may be transformed by normal environmental processes to either increase or decrease the bioavailability of the toxic species…. Nevertheless, the concepts that a substance…may not be rapidly lost from the environment…are as applicable to metals and metal compounds as they are to organic substances" (United Nations 2017).

Furthermore, only initial guidance is available on how to assess the “degradability” of metals (GHS, section A9.7.1.7). Aspects that should be considered include: evidence of bioavailability change over time, metal ion partitioning from the water column via naturally occurring processes, information on residence time of metals in the water column, and evidence on the processes involved at the water–sediment interface, including deposition and remobilization (United Nations 2017).

Skeaff et al. (2002) proposed an approach to assessing metal "degradability" in the context of loss from the water column using metal "half‐times" (analogous to degradation half‐life) as a metric. This approach also considered the permanence of removal by looking at the extent of metal remobilization from sediment. Their work focused on analysis of field data, and they called for the development of a standard set of laboratory conditions to assess partitioning half‐times, although no details were offered as to the nature and setup of such bench‐scale tests. A logical extension of the Skeaff et al. (2002) approach is to consider 70% removal times (in addition to half‐times) relative to 28 d because GHS degradability assessments consider this removal extent and time frame (United Nations 2017).

The goal of the present review was to assess the fate of copper added to surface water. The intrinsic chemical properties of copper were examined in the context of environmental fate. Similar to Skeaff et al. (2002), data on the fate of copper added to lakes, microcosms, and mesocosms were compiled and analyzed to determine copper removal rates from the water column. Studies on copper behavior in sediment were also reviewed to assess the potential for remobilization. To supplement existing field data, a previously developed, screening‐level fate and transport model (tableau input coupled kinetic equilibrium transport–unit world model [TICKET–UWM]) was parameterized and applied to quantify copper removal rates and remobilization in a standardized lake setting. Field and modeling results were reconciled, to provide a more complete understanding of the fate of copper added to surface water. This information may be useful in the context of environmental hazard assessment of copper.

## FATE OF COPPER ADDED TO SURFACE WATER: INTRINSIC PROPERTIES

Metals, by virtue of their unique chemical properties, are distinct from other elements and compounds. Trace metals such as copper, zinc, and lead possess an intrinsic propensity to react in the environment in ways that influence their bioavailability and fate (Pearson 1963). Copper, for example, interacts strongly with various functional groups present in the following: natural organic matter, including dissolved organic carbon (DOC) and particulate organic carbon (POC); iron oxides, including hydrous ferric oxide (HFO) and goethite; and manganese oxides, including hydrous manganese oxide (HMO; Flemming and Trevors 1989; Dzombak and Morel 1990; Tipping and Hurley 1992; Tonkin et al. 2004).

These interactions are largely responsible for copper's strong affinity for particulate matter and relatively large distribution coefficient (*K*_D_). The solubility of copper in the environment can be limited through precipitation of copper hydroxides/oxides, carbonates, and, perhaps most importantly, sulfides. The ability of a given trace metal, such as copper, to form precipitates is described quantitatively with a solubility product or *K*_sp_. Solubility products for each metal are unique and vary considerably between metals (Di Toro et al. 2001b). Copper inherently forms highly insoluble sulfide precipitates in sulfidic environments such as anoxic sediment (Dyrssen and Kremling 1990; Stumm and Morgan 1996). Moreover, copper has been shown to have a higher affinity for sulfide than other trace metals such as nickel, cadmium, and zinc (Berry et al. 1996). As will be discussed below in the review of copper remobilization from sediment, the precipitation of copper sulfide solids and binding to organic matter and iron oxides limit the bioavailability of copper. Affinity for particles and participation in reactions that sequester metal and/or limit bioavailability are critical to evaluation of removal from the water column and remobilization potential, as further discussed below in the review of field studies and modeling analysis. Although it is true that environmental conditions in the water and sediment (e.g., pH and redox conditions) can impact the surface complexation and precipitation reactions listed above, the tendency of copper to participate in these reactions is inherent to copper.

The copper complexation, adsorption, and precipitation reactions discussed above all involve formation/breaking of bonds with significant covalent character and, therefore, constitute material changes in speciation. These changes in speciation can reduce copper bioavailability (Di Toro et al. 2001a). The bond formation/breaking associated with speciation changes separates copper (and other metals) from organic substances. Although organic substances can adsorb to DOC and POC (e.g., in suspended particles), they do not undergo the same types of complexation and precipitation reactions. Adsorption of organic chemicals to DOC and POC occurs primarily via relatively weak van der Waals attractive forces (Schwarzenbach et al. 1993). These interactions do not involve covalent bond formation/breaking. The physical and chemical properties of the organic chemical are fundamentally unchanged during this process. Thus, although organic substances can adsorb to settling particles and be transported to the sediment, there is no fundamental change in speciation, as is the case for copper and other metals. Metal removal involves formation/breaking of bonds with a significant covalent nature in an analogous fashion to the formation/breaking of covalent bonds in organics during degradation. The result of metal removal and organic degradation is similar: reduction in the potential for toxicity.

## FATE OF COPPER ADDED TO SURFACE WATER: FIELD AND LABORATORY STUDIES

### Studies on removal of copper from the water column

When we assembled field and laboratory studies examining the loss of copper from the water column, emphasis was placed on studies in which copper was added to the water column in a "spike" load. Data from these types of studies are most useful in the context of degradability and hazard assessment. For a spike load of a particle reactive metal such as copper, the general response is an instantaneous increase in concentration followed by a decline in concentration as copper is lost from the water column via adsorption/precipitation/sedimentation processes, diffusion into the sediment, and washout. A single field study with a continuous load of copper was assessed. For a continuous copper load into a flow‐through system, the expected water column response is an increase of copper from the initial concentration to some steady‐state concentration. Whole‐lake studies are presented first, followed by microcosm and mesocosm studies.

#### Whole‐lake studies

Effler et al. (1980) examined the impact of copper sulfate treatment on Cazenovia Lake, a hard water (hardness = 100–140 mg/L CaCO_3_), alkaline (pH = 7.6–8.55), mesotrophic lake in the village of Cazenovia (NY, USA) with an average depth of 7.9 m. The lake was treated with copper sulfate (as CuSO_4_·5H_2_O) starting in 1964. The lake was monitored following 3 copper sulfate treatments of 455 kg of CuSO_4_·5H_2_O in July, August, and September of 1977. Total copper concentrations immediately following these 3 applications were approximately 9.2, 14, and 7.9 µg/L, respectively. Within 23 d of the applications, total copper concentrations declined to levels approximately 34 to 68% lower than the concentrations at dosing.

Haughey et al. (2000) assessed the fate of copper added to Lake Mathews, a drinking water reservoir in Riverside, California (USA). They sampled the lake before and after an addition of copper sulfate (as CuSO_4_·5H_2_O) in late October 1995. The peak total copper concentration immediately after dosing was 17 µg/L. Copper added to the water column was rapidly converted to particulate forms and transported to the sediment. Approximately 62% removal of total copper was observed within 9.4 d of dosing. Haughey et al. (2000) calculated that only 20% of the copper was exported from the reservoir over the 70 d following dosing.

van Hullebusch and colleagues assessed the fate of copper in 2 shallow lakes in the Limousin region of France treated with copper sulfate to control algal blooms (van Hullebusch et al. 2002, 2003a, 2003b). In June 2000, Lake Courtille in Guéret (average depth = 1.77 m) was dosed with both aluminum sulfate and copper sulfate (CuSO_4_∙5H_2_O) to control algal populations (van Hullebusch et al. 2002). In June 2001, copper sulfate was added to the reservoir in Saint Germain les Belles (average depth = 1.6 m; van Hullebusch et al. 2003a, 2003b). Total and dissolved copper in the water column was monitored following copper addition.

**Figure 1:**
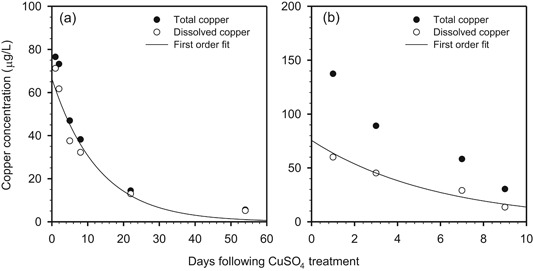
Time series of total (closed circles) and dissolved (less than 0.45 µm, open circles) copper concentrations in the water column of (a) Lake Courtille and (b) Saint Germain les Belles Reservoir. Note: the *y*‐ and *x*‐axes scale differ between the 2 panels. The solid line is a first‐order loss (i.e., *C* = *C*_0_ exp[–kt]) fit through the dissolved copper data.

For both lakes, water column copper concentrations declined rapidly (Figure 1). For example, in Lake Courtille, the dissolved copper concentration on day 22 was more than 80% lower than that measured on day 1. Similarly, for the Saint Germain les Belles Reservoir, the dissolved copper concentration on day 9 was more than 75% lower than that measured on day 1. First‐order loss fits to the data (lines in Figure 1) indicated that the time required for 50% removal (i.e., half‐time) was approximately 9 d for Lake Courtille and approximately 4.1 d for Saint Germain les Belles Reservoir. The contribution of dilution to the water column half‐time was assessed using the hydraulic detention time because this parameter was available for the 2 lakes. The hydraulic detention times for Lake Courtille and Saint Germain les Belles Reservoir are more than 1 yr and 0.25 yr, respectively. The calculated half‐times associated with dilution are approximately 250 d for the lake and 63 d for the reservoir. These values are considerably larger than the half‐times observed from the data. This finding indicates that dilution from inflow was not a major metal removal process in these systems. The primary removal mechanism was likely loss to the sediments via settling and/or diffusion.

Liu et al. (2006) assessed the fate of copper applied to catfish ponds as an algaecide. Results from a pilot‐scale experimental pond (29 m long × 14 m wide × 0.8–0.9 m deep) indicated that greater than 90% of the copper added to the water column became strongly sorbed to particles within 2 h. Within 2 d of dosing, more than 99% of the copper had been transferred to the sediment. Approximate times to reach 70% removal were 0.88 d for dissolved copper and 0.84 d for total copper. Based on a mass balance analysis, Liu et al. (2006) found that essentially all the copper added to the ponds ended up in the sediment, where bioturbation helped transport it to the deeper sediment layers.

#### Microcosm and mesocosm studies

**Figure 2:**
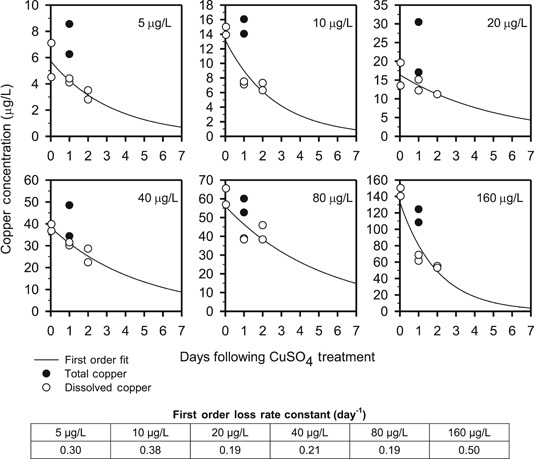
Time series of measured total (closed circles) and dissolved (less than 0.45 µm, open circles) copper in microcosms at various nominal copper concentrations. Plotted data are the arithmetic average of measurements from duplicate microcosms. The solid line is a first‐order loss (i.e., *C* = *C*_0_ exp[–kt]) fit through the dissolved copper data.

A microcosm study was undertaken at the Fraunhofer Institute for Molecular Biology and Applied Ecology (Schmallenberg, Germany) to study the effects of continuous copper exposure on aquatic organisms (Schäfers 2001). A series of microcosms (110 cm long × 96 cm wide) were prepared with a 15‐ to 20‐cm‐deep sediment layer and an overlying water column of approximately 750 L. The depth of the water column was 0.76 m. The microcosms were dosed with copper sulfate to achieve 6 nominal concentrations of copper: 5, 10, 20, 40, 80, and 160 µg/L. The experimental setup included duplicates (i.e., 2 microcosms for each nominal copper concentration). Additional details on that study are provided in the Supplemental Data. Samples for dissolved and total copper analysis were taken after dosing. The dissolved copper data indicated a marked decrease over time (Figure 2). The decrease was significant enough that the mesocosms had to be repeatedly dosed with copper to maintain the desired concentrations. Estimated times for 50 and 70% removal of dissolved copper ranged between 1.4 and 3.7 and between 2.4 to 6.3 d, respectively. The microcosms were not flow‐through systems, so the observed removal was not attributable to washout. Li et al. (2012) also had difficulty maintaining copper concentrations in their experiments because of loss from the water column.

Smolyakov and colleagues investigated the fate of copper and other trace metals in surface water using 3‐m‐deep mesocosms mounted near the shore of the Novosibirskoye Reservoir in Novosibirsk, Russia (Smolyakov et al. 2010a, 2010b). The reservoir water was considered moderately hard (Durfor and Becker 1964 1962), based on a hardness of approximately 70 mg/L as CaCO_3_ (Smolyakov et al. 2010a, 2010b). The suspended particulate matter (SPM) concentration and pH values were approximately 15 mg/L and 8.5, respectively. In one study, 2 separate mesocosms—one exposed to sunlight and one kept in the dark—were dosed with Cu(NO_3_)_2_ to achieve initial an initial copper concentration of 250 µg/L (Smolyakov et al. 2010b). After 20 d, the mesocosms were dosed a second time to once again achieve a copper concentration of 250 µg/L. Copper removal in both the light and dark mesocosms was rapid. After 19 d, dissolved copper concentrations decreased by 95% (light) and 86% (dark). Removal rates for the second dosing were generally similar (Smolyakov et al. 2010b). Comparable rapid copper removal was observed in a companion study with a higher copper loading of 1000 µg/L and with added floating aquatic plants (water hyacinth; Smolyakov et al. 2010a). Uptake of copper by these plants may have contributed to the copper removal. Given the elevated water pH and total copper concentrations associated with the companion study, it is likely that some fraction of the particulate copper in the water column was precipitated copper hydroxide or copper oxide. Speciation calculations made using water chemistry data provided in Smolyakov et al. (2010a) and Smolyakov et al. (2010b) confirmed that the solubility product of copper (hydr)oxide was exceeded. This result highlights the concept that direct precipitation of trace metals (e.g., as hydroxides, oxides, and carbonates) in addition to sorption, can transfer metal to the particulate phase and result in loss from the water column via settling.

The MEtal LIMnological Experiment (MELIMEX) study was undertaken to assess the effects of increased metal loading (relative to natural levels) on lacustrine biota, to determine the accumulation and distribution of metals in the food chain, and to investigate the speciation, distribution, and fate of added metals (Gächter 1979). The experiment was conducted in Lake Baldegg (Lucerne, Switzerland) using large enclosures called limno‐corrals (12 m in diameter and 10 m deep) to isolate portions of the lake water column and sediment for study. From April 1977 to June 1978, copper was added continuously to 2 of the 3 limno‐corrals (1 served as a control) via inflow to the enclosures at a concentration of 180 nM (11.4 µg/L; Gächter 1979). Periodically during the study, the water column was sampled for several water quality parameters including dissolved copper.

**Figure 3:**
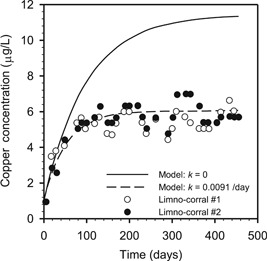
Time series of dissolved copper data from the MELIMEX enclosure study (Gächter 1979). Data points are measured data from Gächter and Geiger (1979). The lines are model results reproduced from Di Toro et al. (2001b) with and without a first‐order loss process included.

As expected for continuous copper addition in a flow‐through system, dissolved copper increased asymptotically to a steady‐state water column concentration of approximately 6 µg/L (Figure 3). Model results from Di Toro et al. (2001b) with (dashed line) and without (solid lines) a first‐order loss reaction clearly show the impact of metal removal processes. The steady‐state water column concentration with removal (~6 µg/L) was almost half the value without removal (~11 µg/L). The first‐order loss rate constant of 0.0091 d^–1^ corresponds to a half‐time of approximately 76 d.

#### Summary of removal times from field studies

Table 1 summarizes the water column copper removal from the whole‐lake, microcosm, and mesocosm studies in terms of 50 and 70% removal times. When possible, a first‐order loss rate was calculated from the concentration versus time data and used to calculate the removal times. The calculated 50% removal times ranged between 0.1 and 76 d. The calculated 70% removal times ranged from approximately 0.8 to 130 d. Removal was relatively rapid. For the majority of studies examined, more than 70% of the added copper was removed from the water column within 16 d of dosing. One exception is the MELIMEX experiment. This lake was characterized by a very low particle settling velocity of 0.2 m/d (more than 10 times below the value used in the European Union system for the evaluation of substances (EUSES) generalized lake described in the *Application of the TICKET*–*UWM to the EUSES model lake: Water column* section), a low suspended solids concentration (5.9 vs 15 mg/L in EUSES), and a low copper concentration in the inflow (11.4 µg/L).

**Table 1:** Summary of estimated 50% and 70% removal times

System^a^	Sample fraction	50% Removal time (d)^b^	70% Removal time (d)^b^
Cazenovia Lake	Total		
July 1977 addition		9.6	70% removal not observed in 23 d
August 1977 addition		2.0	14.1
September 1977 addition		50% removal not observed in 22 d	70% removal not observed in 22 d
Lake Mathews	Total	15.6 (9.2–52.3)	27.2 (16.0–90.8)
Lake Courtille	Dissolved	9.0 (6.5–14.7)	15.6 (11.2–25.6)
St. Germain les Belles Reservoir	Dissolved	4.1 (2.2–28.3)	7.1 (3.8–49.1)
Catfish pond	Dissolved	0.11	0.88
	Total	0.48 (0.38–0.66)	0.84 (0.66–1.1)
IME microcosms^c^	Dissolved		
5 µg/L		2.3 (1.2–15.5)	4.0 (2.2–26.9)
10 µg/L		1.8 (1.1–6.1)	3.1 (1.8–10.6)
20 µg/L		3.6 (1.3–na)^d^	6.3 (2.3–na)
40 µg/L		3.3 (2.0–8.8)	5.7 (3.5–15.3)
80 µg/L		3.7 (1.6–na)	6.3 (2.7–na)
160 µg/L		1.4 (0.90–3.0)	2.4 (1.6–5.2)
Novosibirskoye Reservoir mesocosms^e^	Dissolved
Dark, 1st addition		7.5 (7.1–7.8)	13.0 (12.4–13.6)
Dark, 2nd addition		7.3 (6.8–7.8)	12.7 (11.9–13.6)
Light, 1st addition		5.0 (4.6–5.3)	8.6 (8.0–9.3)
Light, 2nd addition		4.5 (4.0–5.2)	7.8 (6.9–8.9)
Dark, 1st addition	Total	8.7 (8.6–8.9)	15.1 (14.9–15.4)
Dark, 2nd addition		8.3 (8.0–8.8)	14.5 (13.8–15.2)
Light, 1st addition		5.8 (5.6–5.9)	10.0 (9.8–10.2)
Light, 2nd addition		6.2 (6.0–6.5)	10.8 (10.4–11.3)
MELIMEX mesocosms	Dissolved	76.2	130
TICKET‐UWM (empirical *K*_D_)	Dissolved	2.7	4.7
TICKET‐UWM (calculated *K*_D_)	Dissolved	1.6	2.7

^a^ Sources: Lake Courtille and St. Germain les Belles Reservoir: van Hullebusch et al. (2002, 2003a, 2003b); IME Mmcrocosms: Schäfers (2001); Novosibirskoye Reservoir: Smolyakov et al. (2010a, 2010b); Lake Matthews: Haughey et al. (2000); catfish ponds: Liu et al. (2006); Cazenovia Lake: Effler et al. (1980); MELIMEX: Di Toro et al. (2001b); Gächter (1979); TICKET‐UWM: present study.

^b^ Numbers in parentheses denote the 95% confidence interval of 50 or 70% removal times. This interval was developed from the 95% confidence interval of the first‐order loss rate constant fit to the copper concentration data for each study. For the catfish pond dissolved copper data and Cazenovia Lake total copper data, application of a first‐order model was not supported. Removal time values were interpolated from plots of fraction remaining versus time. Removal times for MELIMEX were calculated from the first‐order loss rate constant of 0.0091 day^‒1^ determined by Di Toro et al. (2001b). No information was provided on the uncertainty associated with this rate constant.

^c^ Removal times were determined for each nominal copper dosing concentration in the IME microcosms.

^d^ Upper 95% confidence limit of the first‐order rate constant was positive, indicating an *increase* in copper. An increase in dissolved copper concentration between consecutive samples occurred in the 20‐ and 40‐µg/L test systems but for only 1 of the 2 duplicates.

^e^ Removal times were determined for each treatment (light, dark) and copper addition (first and second) separately in the Novosibirskoye Reservoir study.

IME = Fraunhofer Institute for Molecular Biology and Applied Ecology (Schmallenberg, Germany); MELIMEX = MEtal LIMnological Experiment; TICKET–UWM = tableau input coupled kinetic equilibrium transport–unit world model; na = not available.

### Studies on remobilization of copper from sediments

The literature just summarized indicates rapid loss of soluble copper from the water column to sediments, where, based on its intrinsic properties, copper can precipitate with sulfide under anoxic conditions. A complete analysis of copper fate requires consideration of the potential for copper to be remobilized from sediment. One important potential remobilization mechanism is oxidative release of copper from sulfide phases in sediment when oxic conditions are encountered. This can happen through larger scale resuspension events (e.g., currents, dredging, and other disturbances) or through bioturbation (where the action of filter‐feeding and surface‐scavenging organisms mixes porewater and resuspends sediment). Studies on copper remobilization were reviewed by Skeaff et al. (2002; Supplemental Data, Table SI‐1). Although some remobilization of copper was indicated in certain studies, Skeaff et al. (2002) concluded that the net flux of metals was generally directed toward the sediment. Data from additional studies described in the *Acid‐volatile sulfide oxidation, Changes in redox conditions and the effect on bioavailability*, and *Resuspension events* sections provide more insight into the potential remobilization of copper from sediments.

### Acid‐volatile sulfide oxidation

Simpson et al. (1998) conducted 8‐h oxidation studies with synthesized amorphous metal sulfides ("model metal sulfide phases") and resuspension experiments with natural sediment. Metal concentrations (including copper) were assessed using bulk sediment measurements of acid‐volatile sulfide (AVS) and simultaneously extracted metal (SEM). The AVS/SEM procedure involves an acid extraction with hydrochloric acid. Their oxidation study results indicated that the amount of SEM(Cu) extracted from model copper sulfides—interpreted as oxidative release—was only 20% for CuS and 40% for Cu_2_S. This finding provides evidence that pure copper sulfide phases possess resistance to oxidative release of copper. In the resuspension experiments with natural sediment, greater amounts of SEM(Cu) were observed. However, the author concluded that the observed increase in SEM(Cu) over the duration of the resuspension experiments with natural sediment was not indicative of release due to oxygenation of the system. They suggested that the SEM(Cu) increase was due to the presence of iron phases in the natural sediment and an analytical artifact associated with an Fe^3+^‐catalyzed oxidation mechanism that was favorable under the low pH conditions with the SEM extraction procedure. The authors also concluded that iron sulfide (FeS) and manganese sulfide (MnS) phases, if present in excess over trace metal sulfide phases (e.g., Cu‐, Cd‐, Ni‐, Pb‐, and Zn‐sulfides), can buffer bioturbation‐induced trace metal sulfide oxidation. They stated that trace metals associated with FeS may be released after oxidation of the FeS carrier phase. However, they concluded that additional studies are required to assess the potential for metal release during short‐term exposure to oxic conditions. Several studies in the following two sections address this issue.

### Changes in redox conditions and the effect on bioavailability

Sundelin and Eriksson (2001) studied the mobility, bioavailability, and toxicity of metals in sediment from the inner archipelago of Stockholm (Sweden). Specifically, they investigated the influence of oxygenation and bioturbation on metal mobility and bioavailability using metal‐contaminated sediments. Sediments were incubated in a flow‐through system with brackish water (7‰) for 3 to 7 mo both with and without bioturbation. They observed decreases in total sulfur, which they attributed to the oxidation of sulfides as the sediment samples were exposed to oxic overlying water. Sediment copper concentrations remained constant in the surface sediment despite the effects of oxygenation and bioturbation. Furthermore, copper levels in the outflow water from the incubation chambers were not elevated compared with controls. Copper was not bioaccumulated in amphipods added to the experimental systems. These finding provide evidence that in natural, unspiked sediments, copper release from sulfide and other sediment binding phases during exposure to oxic conditions is very limited.

van der Geest and León Paumen (2008) exposed soils from 2 different sites (1 contaminated and 1 uncontaminated) from a floodplain on the River Waal (The Netherlands) for a period of 10 wk to continuous flow of natural river water under both depositional and erosional conditions. Changes in redox conditions (oxidation‐reduction potential measurements), copper availability (diffusive gradient film measurements), and copper uptake by organisms were quantified, and then colonization, succession, and functioning of the microphytobenthic community were evaluated. The authors found no simple relationship between sediment redox state and bioavailability within a vertical profile of each sediment. The contaminated sediment did not show marked signs of impairment with respect to indicators such as colonization, growth, and succession of algal communities. Accumulation of copper in *Tubifex* worms was only slightly different from levels in worms placed in the uncontaminated sediment. The study of van der Geest and León Paumen (2008) provides an example of how low copper bioavailability can exist following significant changes in redox status.

Teuchies et al. (2011) assessed the impact of increased dissolved oxygen concentrations on copper and other metal dynamics in the Zenne River in Belgium. The goal was to determine whether higher dissolved oxygen resulting from the initiation of wastewater treatment plant operations could turn the sediments into metal sources as opposed to sinks. In experimental setups mimicking anoxic, oxic, and oxic with high turbidity systems, they observed lower AVS in the oxic systems compared with the anoxic system and significantly higher dissolved copper in the oxic systems relative to the anoxic system. However, sulfide oxidation and copper release were not readily observable in the field despite the large increase in dissolved oxygen associated with the start‐up of the wastewater treatment plant. In fact, based on available data from a station on the Zenne River, there was an increased frequency of nondetects for dissolved copper after the plant start‐up in March 2007 (Flanders Environment Agency 2016). Dissolved copper was below the detection limit for all samples taken at this station from 2010 through 2014. Copper release from sediment was not able to sustain indefinitely the elevated water column concentrations occurring before wastewater treatment began. Slow oxidation rates and/or high dilution by river water were mentioned by the authors as ways metal remobilization were obscured in the field relative to what was observed in the laboratory.

A pair of recent studies assessed copper behavior in sediments exposed to an oxic water column (De Jonge et al. 2012; Costello et al. 2015). De Jonge et al. (2012) monitored changes in redox status, sediment geochemistry, and metal bioavailability over a 54‐d period in experimental flow‐through set‐ups containing metal‐contaminated sediment and overlying surface water with dissolved oxygen at approximately 40 or 90% of saturation. Over the study period, the oxidation‐reduction potential of the upper sediment layer increased markedly, and the AVS decreased by 70% in the treatment with high oxygen. Labile copper concentrations in porewater did not increase over time, which supports the conclusion that it remained bound to the sediment. Furthermore, after 54 d, no significant release of copper to the overlying water was found. The authors attributed the limited metal release to the overlying water to the presence of remaining excess AVS and additional metal binding by iron and manganese (hydr)oxides and organic carbon.

Costello et al. (2015) studied copper dynamics during sediment aging. Sediments from the Ocoee River (Benton, TN, USA) and Dow Creek (Midland, MI, USA) were spiked with copper, incubated for 28 d under a nitrogen atmosphere, and then aged for 213 d in recirculation flumes where the water column was well oxygenated (8.2 mg/L at 23 °C). Over the course of the study, oxygen penetration into the sediment was evident, and AVS declined in the surface layer of the sediment that initially had high AVS. Although the total amount of sediment copper did not change during the study, the porewater and labile copper concentrations decreased rapidly. The authors attributed the loss of porewater copper to binding to iron oxides. They also noted a shift in copper associated with HFOs to more crystalline ferric oxides. In general, older sediment was found to be less toxic than freshly spiked sediment. Finally, the authors found that very little copper was released from the sediment to the overlying water. Copper efflux rates from sediment range from 2.2 to 20 µg/m^2^/h (Ocoee River) and from 4.4 to 57 µg/m^2^/h (Dow Creek). The corresponding amount of copper lost from the sediment on an annual basis was less than 0.7% for the Ocoee River sediment and less than 3% for the Dow Creek sediment.

Haughey et al. (2000) also estimated the rate of copper release from the sediment in their Lake Mathews study. The rate was 620 µg/m^2^/d or 26 µg/m^2^/h. This was orders of magnitude lower than indicated in batch desorption kinetic experiments conducted with sediment. It is, however, generally consistent with the efflux rates observed by Costello et al. (2015). Based on the observation that a significant portion of sediment copper was associated with the oxidizable and carbonate‐bound form, the authors concluded that significant release of copper from the key binding phases in sediment would require large changes in pH and redox conditions.

### Resuspension events

van den Berg et al. (2001) assessed the potential for dredging‐related mobilization of metals in a freshwater tidal marsh area in the lower delta of the rivers Rhine and Meuse in The Netherlands. The authors found that, although the release of dredge spoils to the water column was manifested as an observable increase in the metal content of the SPM, the dissolved metal concentrations in the water column were not markedly affected by the dredging activities.

Fetters and colleagues studied mobilization of zinc, copper, cadmium, lead, nickel, and chromium during short‐term (i.e., 4‐h) resuspension events for both freshwater and marine sediments (Fetters 2013; Fetters et al. 2016). Metal‐contaminated sediment from Lake Depue in Illinois (USA) and Portsmouth Naval Shipyard in Maine (USA) were resuspended in sediment flux exposure chambers. Sediments from both locations had elevated copper levels, with concentrations ranging between 364 and 575 µg/g. After resuspension, dissolved copper concentrations in the water column of the Lake Depue experiment remained below the practical limit of quantification of 8 µg/L (i.e., for the inductively coupled plasma–optical emission spectrometry instrument used for freshwater sample analysis). In the Portsmouth Naval Shipyard experiments, resuspension resulted in minimal release of dissolved metals. For Portsmouth Naval Shipyard sample MS03, dissolved copper was detected in the *t* = 0 h water column sample at a concentration of 4 μg/L. It then dropped to less than 2.67 µg/L (i.e., the method detection limit of the Calscience analysis for Portsmouth Naval Shipyard saltwater samples) for the other 2 samples taken during the 4‐h resuspension event. For Portsmouth Naval Shipyard sediment sample MS04, dissolved copper was not detected in the water column during the 4‐h resuspension event.

## FATE OF COPPER ADDED TO SURFACE WATER: A MODEL STUDY

### Description of the model

Unit world models (UWMs) are screening‐level models used to assess the fate and effects of chemicals through simultaneous consideration of chemical partitioning, transport, reactivity, and bioavailability (MacKay 1979, 1991; MacKay and Paterson 1991). A UWM for metals (TICKET–UWM) has been developed that includes a detailed quantification of metal speciation, bioavailability, and transport (Farley et al. 2011). The TICKET–UWM can be used to assess long‐term fate and effects of metals in the environment. The model domain consists of a single oxic water column layer and a single sediment layer. Additional details on TICKET–UWM are provided in the Supplemental Data.

### Application of TICKET–UWM to the EUSES Model Lake: Water column

Application of fate and transport models, such as the TICKET–UWM, allows for detailed examination of the chemical and physical processes responsible for metal transport and fate under standardized conditions. Once established, the standardized test environment or generalized lake can be further used to examine parameter sensitivities and compare removal and remobilization rates among different metals and other substances.

A generalized lake scenario based on the EUSES regional model (European Commission 2004) was developed to assess metal 50% removal times (i.e., half‐times) and 70% removal times and remobilization potential in a standardized environment. Hereafter this generalized lake will be termed the EUSES Model Lake. The physical and chemical parameters of the EUSES Model Lake were based on GHS transformation/dissolution testing protocols (United Nations 2017) and the EUSES model (European Commission 2004). Additional details regarding model parameters are provided in the Supplemental Data.

**Figure 4:**
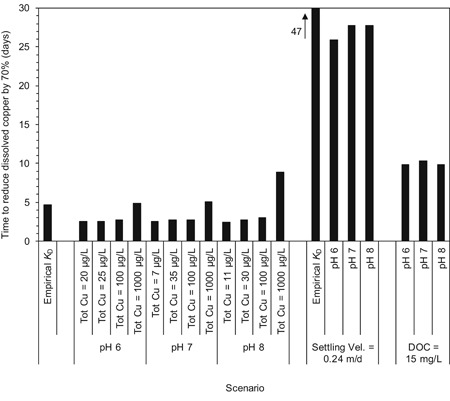
Results of European Union system for the evaluation of substances (EUSES) Model Lake water column analysis. Results from simulations using the Empirical *K*_D_ method are labeled as such. All other results are from simulations using the calculated *K*_D_ method. Vel. = velocity; DOC = dissolved organic carbon.

Metal sorption to SPM was described in the EUSES Model Lake simulations using 2 separate methods. For the first method, water and sediment distribution coefficient (*K*_D_) values were specified based on empirical data from a copper partition coefficient review (Heijerick and Van Sprang 2005). These empirical *K*_D_ values are 10^4.48^ L/kg for the water and 10^4.39^ L/kg for the sediment. For the second method, separate water and sediment *K*_D_ values were calculated with Windermere humic aqueous model (WHAM) VII at each time step in TICKET–UWM simulations (calculated *K*_D_ values). This second method allows one to consider the impact of water chemistry and metal loading on *K*_D_ and copper fate. Simulation results for both methods are shown in Figure 4. For the simulations with the empirical *K*_D_ values, total and dissolved copper concentrations decreased by 50% approximately 2.7 d after copper addition and by 70% approximately 4.7 d after dosing (Figure 4 and Supplemental Data, Figure SI‐2). After 28 d, the dissolved copper concentration in the water column was 0.22 µg/L which is approximately 160 times lower than the starting concentration of 35 µg/L. For the calculated *K*_D_ simulation at pH 7 with the same initial copper concentration, log *K*_D_ values varied between 4.85 and 4.88 in the water column. WHAM VII predicted that more than 51% of the copper would be bound to particles. This is somewhat greater than the value from the empirical *K*_D_ simulation of 31%. Consequently, copper removal from the water column was faster than in the empirical *K*_D_ simulation (Figure 4 and Supplemental Data, Figure SI‐2). Dissolved copper concentration decreased by 70% in 2.7 d. Detailed model output from these simulations is presented in the Supplemental Data, Tables SI‐4 and SI‐5. Model output for the empirical *K*_D_ and calculated *K*_D_ values are also included in the removal time summary in Table 1.

Sensitivity analysis simulations were performed to assess: 1) the effect of copper loading with initial total copper concentrations at the 2 chronic classification cutoff values of 0.1 and 1 mg/L (European Chemicals Agency 2017); 2) the effect of water chemistry using the pH 6, 7, and 8 water chemistries from the GHS transformation/dissolution testing protocols (United Nations 2017; see the Supplemental Data for details); 3) the impact of decreasing the settling velocity from the EUSES Model Lake value of 2.5 to 0.24 m/d (lower end of the POC range from Burns and Rosa 1980); and 4) the impact of increasing DOC from 2 to 15 mg/L to reflect the approximate 10:1 DOC:POC ratio found in some lakes (Wetzel 2001).

Model‐predicted times to reduce initial dissolved copper concentrations by 70% varied to a limited extent (range = 2.5–8.9 d) in the simulations in which initial total copper concentration and water chemistry were varied (Figure 4). Model‐predicted 70% removal times increased with an increase in DOC from 2 to 15 mg/L, but the change was relatively small. The formation of copper–DOC complexes reduced the distribution coefficient, decreased the fraction of copper sorbed to particles, and, consequently, reduced the rate of water column copper removal via settling. The increase in removal times for decreased settling velocity simulation (i.e., 2.5–0.24 m/d) was relatively large compared with the changes associated with the variation of other parameters (Figure 4). The maximum 70% removal time in the sensitivity analysis of 47 d occurred for the scenario using the empirical *K*_D_ value and a settling velocity of 0.24 m/d. Detailed model output from the sensitivity analysis simulations is presented in the Supplemental Data, Tables SI‐5, SI‐6, and SI‐7.

### Application of TICKET–UWM to the EUSES Model Lake: Sediment

Research has shown that the reaction of copper with sulfide in the form of AVS to form insoluble copper sulfide is a key process that mitigates the bioavailability and toxicity of copper in sediments and influences its fate in natural systems (Berry et al. 1996; Di Toro et al. 1992, 2001b). Settling represents the primary metal source to the sediment. Resuspension and diffusion are transport processes associated with metal remobilization from the sediment to the water column. In the model, burial of metal from the active sediment layer permanently removes copper from the system.

A series of model simulations was performed to assess copper speciation in sediment and the potential for copper remobilization from the sediment to the water column. Sediment simulations were 1 yr in duration and examined various chemistries and redox conditions (see Supplemental Data, Table SI‐8 and the associated text for details of the sediment simulation approach and parameters used). These simulations considered 2 forms of copper‐sulfide solids (see Supplemental Data).

### Anoxic sediment simulations

Based on model results from day 20 of the simulation, almost all copper in the sediment was found to be present in the particulate phase. Of the very small amount of dissolved copper in the porewater, more than 99% of it was present as copper–DOC complexes. “Free” copper (Cu^2+^) made up less than 0.1% of the dissolved copper. Particulate copper speciation in the sediment simulation was dominated by the copper‐sulfide solid CuS(s). A more detailed summary of the speciation data is provided in the Supplemental Data, Table SI‐9.

**Figure 5:**
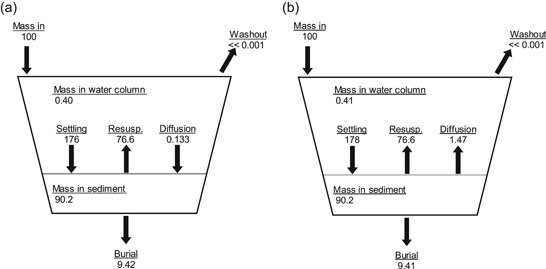
Mass balance results for tableau input coupled kinetic equilibrium transport–unit world model (TICKET–UWM) sediment simulations with an (a) anoxic sediment and (b) oxic sediment. Mass values from the model have been normalized to an input mass of 100 kg. Simulations for both sediment types had an initial total water column copper concentration of 35 μg/L. The anoxic sediment simulation had an acid‐volatile sulfide = 9.1 μmol/g. The oxic sediment simulation had sediment hydrous ferric oxide (HFO) = 18 600 mg HFO/kg and sediment hydrous manganese oxide (HMO) = 154 mg HMO/kg.

At the end of the 1‐yr simulation, greater than 99% of the copper added to the system was in the sediment or had been buried as CuS(s) (Figure 5a). The magnitude of the settling, resuspension, and diffusion terms indicates that, based on the transport parameters in the model (Supplemental Data, Table SI‐8), copper mass transfer between the water column and sediment was dominated by transport on solids. Diffusion played a minor role. but, integrated over the 1‐yr simulation (hereafter termed the integrated diffusive flux), it was directed into the sediment layer. This was a consequence of the highly insoluble nature of the CuS(s) precipitate and the associated very low porewater copper concentration. Resuspension did transfer copper back to the water column, but this was counterbalanced by the effect of settling. Simulation results with Cu_2_S(s) as the copper‐sulfide solid were almost identical to those with CuS(s) (i.e., the quantities present in Figure 5a). One difference between the 2 cases is that the model‐predicted sediment log *K*_D_ for Cu_2_S(s) was considerably lower than the value for CuS(s).

### Oxic sediment simulations

Porewater copper speciation was predicted to be dominated by copper–DOC complexes (Supplemental Data, Table SI‐9). Based on model output, greater than 95% of the particulate copper was associated with POC; 4.3% was associated with HFO, and approximately 0.36% was associated with HMO. The model‐predicted log *K*_D_ for the sediment layer of 3.53 was considerably lower than the anoxic case value, which is because no CuS(s) or Cu_2_S(s) was formed in the oxic scenario. A more detailed summary of the speciation data is provided in the Supplemental Data, Table SI‐9.

Mass balance results from the oxic simulations (Figure 5b) were very similar to those for the anoxic simulation (Figure 5a) except for the diffusive flux. The oxic simulation copper diffusive flux was directed out of the sediment, but only resulted in the transfer of 1.5% of the total copper load back to the water column.

### Sensitivity analyses

A series of sensitivity analyses was conducted to assess effect of 1) low AVS (1 μmol/g vs the base case value of 9.1 μmol/g); 2) variation in water column/sediment pH (6/7, 7/7, and 8/7.5 vs the base case combination of 7.07/7.56); 3) low sediment solids concentration (150 g/L_bulk_ vs the base case value of 500 g/L_bulk_); 4) variation in hardness (factor of 2 greater than and less than the base value of 516 mg/L as CaCO_3_); 5) variation in resuspension rate (0.1, 1, 3.2, and 10 times the default rate of 2.44 cm/yr); and 6) variation in copper loading (initial copper concentrations of 10, 100, and 1000 µg/L).

Despite minor differences in the model results, the key findings of greater than 70% removal from the water column in 28 d, sustained dissolved water column concentrations much less than that representing 70% removal, and strong copper binding in the sediment through precipitation as copper sulfide were observed across all sensitivity analysis simulations. The integrated diffusive flux was directed into the sediment, with the exception of the simulation with the highest initial copper concentration and the low AVS concentration. In that simulation, the AVS binding capacity of the sediment was exceeded by the very high copper concentration. A more detailed discussion of these sensitivity analysis simulations can be found in the Supplemental Data.

### Empirical K_D_ simulations

Results from the empirical *K*_D_ simulations indicate total and dissolved copper concentrations at the end of the 1‐yr simulation that were more than 30 times lower than the 70% removal concentration. Although the water column log K_D_ was greater than the sediment log K_D_, the integrated diffusive flux was directed into the sediment.

### Sediment simulation summary

In summary, detailed sediment model simulations indicate that in anoxic sediments, precipitation of copper sulfide significantly enhanced copper binding in the sediment. Consequently, the diffusive copper flux integrated over 365 d was directed into the sediment. Although sediment feedback from resuspension (and diffusion for the oxic sediment simulation) did transfer some copper back to the water column, the resulting concentrations were very low. For all test conditions considered—including oxic sediments and sediments with high resuspension rates—water column copper levels caused by sediment feedback were substantially lower than concentrations corresponding to a 70% decrease from the original dosing concentrations. In other words, sediment feedback did not interfere with long‐term attainment of greater than 70% removal of copper. The detailed sediment model simulations provide evidence that the potential for copper remobilization from sediment was limited and sequestration in the sediment was effectively irreversible.

## SYNTHESIS OF FIELD AND MODEL DATA

### Modeling of the field study at the Saint Germain les Belles Reservoir

Water column copper concentration data from the Saint Germain les Belles Reservoir (van Hullebusch et al. 2003a, 2003b) were used to test the ability of the TICKET–UWM to describe copper fate in lakes. Metal sorption to SPM was described in the model by either 1) specifying a distribution coefficient (*K*_D_) based on measurements in the water column and sediment (empirical *K*_D_ values) or 2) having the model calculate the *K*_D_ value at each time step with a WHAM VII speciation calculation (calculated *K*_D_ values). The water column empirical *K*_D_ was based on measurements in the Saint Germain les Belles Reservoir. The sediment empirical *K*_D_ was based the copper partition coefficient review of Heijerick and Van Sprang (2005). Additional details related to these model simulations can be found in the Supplemental Data.

**Figure 6:**
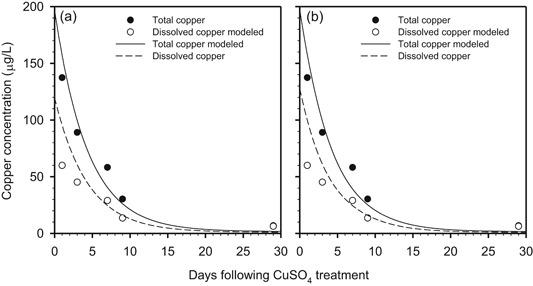
Time series of measured (circles) and modeled (lines) copper in Saint Germain les Belles Reservoir. Model results are from simulation with water column and sediment *K*_D_ values **(a)** specified at the measured/empirical values (surface water: 10^4.56^ L/kg; sediment: 10^4.39^ L/kg) and **(b)** calculated at each time step using a Windermere humic aqueous model (WHAM) VII speciation calculation.

For the empirical *K*_D_ simulation with a log *K*_D_ of 4.56 in the water column, a settling velocity of 0.90 m/d resulted in an optimal fit to the total copper data (Figure 6a). The copper behavior at day 29 was influenced by resuspension caused by heavy rains. Because this acute resuspension event was not included in the model, it is understandable that the model results did not pass through this point.

For the calculated *K*_D_ simulation, water column log *K*_D_ values increased over the 30‐d simulation from 4.49 to 4.57. This highlights the nonlinear, metal‐concentration–dependent sorption predicted by the WHAM speciation model within TICKET–UWM. The model‐calculated log *K*_D_ values were generally consistent with the average measured value of approximately 4.56. As a result, the calibrated settling velocity associated with the calculated *K*_D_ simulation (0.96 m/d) was similar to that from the empirical *K*_D_ simulation (0.90 m/d). These findings for both the empirical and the calculated *K*_D_ simulations with TICKET–UWM indicate that the model can describe copper removal from the surface water with reasonable accuracy.

### Removal of copper from the water column

Water column copper data from lakes, microcosms, mesocosms, and TICKET–UWM simulation provide an expanded picture of copper removal from the water column. A summary of water column removal times, expressed as 70% removal times, is presented in Table 1. The 70% removal times varied from approximately 0.8 to 27 d, with one exception of 130 d. The work of Santschi (1984) provides a means of understanding the variation seen in the removal times. Assuming removal of metals by settling particles in a well‐mixed water column, an expression for the first‐order loss rate (*k*) for total metal (dissolved plus particulate) is as follows:

(1) $$k=\frac{SK_{D}}{H\left(1\;+\;K_{D}m\right)}$$

where *S* is the particle flux (equal to the settling velocity *v*_s_ times the SPM concentration *m*), *K*_D_ is the distribution coefficient (assumed constant), *H* is the average depth, and *m* is the SPM concentration in the water column. The variables in this equation are the key parameters impacting loss from the water column via settling. There are 2 important limiting cases based on the value of *K*_D_ *m*. For *K*_D_ *m* ≫ 1, Equation 1 simplifies to the following:

(2) $$k=\frac{S}{\mathrm{Hm}}=\frac{v_{s}}{H}$$

In this limiting case, essentially all the metal is present on the particles and the metal removal rate depends only on the rate at which particles settle out of the water column via settling. For *K*_D_ *m* ≪ 1, Equation 1 simplifies to the following:

(3) $$k=\frac{SK_{D}}{H}=\frac{v_{s}mK_{D}}{H}$$

Here the metal removal rate depends on the rate at which particles settle out of the water column (*v*_s_/*H*) and the extent to which metal is associated with the particles (*K*_D_ *m* or the ratio of metal on the particles to metal in the dissolved phase).

**Figure 7:**
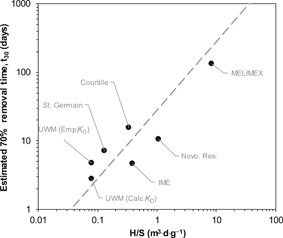
Time required for removal of 70% of the added copper to natural lakes and enclosures, plotted against the ratio of lake depth (H) over the settling flux of suspended particulate matter (S). The dashed line is a theoretical line generated using Equation 4 and average *m* and *K*_D_ values of 10.5 mg/L and 10^4.78^ L/kg, respectively. For IME microcosms, the average of 70% removal times for the 6 nominal copper treatments was used. For the Novosibirskoye Reservoir (Novo. Res.), the average of the 4 dissolved copper 70% removal times is used. Calc = calculated; Emp = empirical; IME = Fraunhofer Institute for Molecular Biology and Applied Ecology (Schmallenberg, Germany); MELIMEX = MEtal LIMnological Experiment; UWM = unit world model.

The solution to the differential equation for first‐order loss of total metal with the rate constant in Equation 1 can be rearranged to provide characteristic removal times (either half‐time or time for 70% removal):

(4) $$t_{x}\;=\;\ln\left(\frac{100}{x}\right)\frac{H\left(1\;+\;K_{D}m\right)}{SK_{D}}$$

(5) $$\begin{array}{cc} t_{x}=\ln\left(\frac{100}{x}\right)\frac{Hm}{S}=\ln\left(\frac{100}{x}\right)\frac{H}{v_{s}} & \text{for}\;K_{D}m\gg 1 \end{array}$$

(6) $$\begin{array}{c} t_{x}=\ln\left(\frac{100}{x}\right)\frac{H}{SK_{D}}=\ln\left(\frac{100}{x}\right)\frac{H}{v_{s}mK_{D}}\;\text{for}\;K_{D}m\ll 1 \end{array}$$

where *x* is the percentage remaining (i.e., 50 for half‐time and 30 for 70% removal). Plots of removal time *t*_x_ versus *H*/*S*—the analog of the plots of Santschi (1984)—are useful in interpreting the data in Table 1. Plots of the 70% removal time (*t*_30_) versus *H*/*S* are shown in Figure 7 for systems that were dosed with copper and for which the information was available to calculate *t*_30_ and *H*/*S*. Particle flux (*S*) data were not available for Lake Mathews, the catfish ponds, or Cazenovia Lake. These waterbodies, therefore, do not appear in Figure 7. For most lakes in Figure 7, the *t*_30_ value was calculated using data on the decrease in copper concentration with time. For the MELIMEX dataset, a theoretical *t*_30_ value was calculated from the first‐order loss rate constant of 0.0091 d^–1^. Also plotted is a theoretical line that was generated from Equation 4 using average *m* and *K*_D_ values from the 7 systems (Supplemental Data).

The removal time data for the lakes generally follow the behavior predicted by Equation 4 (Figure 7). Removal times increase with increasing *H*/*S* values. Intuitively, one would expect that longer removal times would be required for deeper lakes (greater *H*) and lakes with lower particle fluxes to the sediment (lower *S*). Most lakes in the present study, including the TICKET–UWM generalized lake scenarios, have such *H*/*S* values that the time required to attain 70% removal of copper from the water column is less than 30 d. In contrast, the MELIMEX lake is very specific and different from the other lakes, with considerably greater depth and lower particle flux (*S*) values compared with the other lakes (Supplemental Data, Table SI‐13). These observations also set it apart from the TICKET–UWM EUSES Model Lake scenario.

Some of the scatter in *t*_30_ data is caused by differences in metal partitioning behavior (*m* and *K*_D_) between the studied systems. However, it should be noted that the equations in this section were derived considering only metal loss from settling. Diffusion into the sediment and washout are additional processes that serve to decrease metal concentrations in the water column. Furthermore, for shallow systems, direct adsorption to the sediment layer has been indicated as an additional metal removal mechanism (Nyffeler et al. 1986; Bird and Evenden 1996).

### Remobilization of copper from sediments

Collectively, the data assembled on copper behavior in sediments indicate that although release/remobilization of sediment copper to the water column is possible, there are data to support the possibility that the extent and impact of the release is limited. Some studies in marine/estuarine systems have observed metal release, but the overall impact on water column dissolved copper concentrations was minor. Teuchies et al. (2011) observed a release of dissolved copper after aeration in laboratory systems, but the effect was not observable in the field. There has been evidence presented that once copper precipitates with sulfide in sediment, it is not readily released after exposure to oxic conditions (Simpson et al. 1998; Sundelin and Eriksson 2001; De Jonge et al. 2012; Costello et al. 2015). This may be due to the buffering effect of iron and manganese oxides when these are present in excess (Simpson et al. 1998). The work of Costello et al. (2015) highlights the importance of iron oxides as binding phases for copper in oxic sediment. Furthermore, it demonstrates that, whereas the present review focuses on lentic systems, long‐term sequestration of copper also occurs in the sediment of lotic systems. In the extreme short‐term resuspension cases associated with dredging and prop wash events, dissolved copper concentrations in the water column were not markedly affected (van den Berg et al. 2001; Fetters 2013), supporting the conclusion of minimal release of copper to the dissolved phase. The detailed sediment simulations conducted with the TICKET–UWM further indicate minimal copper remobilization from the sediment. Available modeling and field data indicate that copper‐sulfide interactions play a dominant role in copper sediment chemistry. In summary, due to the strong binding of copper in sediments, oxidation of anoxic sediments or resuspension events do not generally lead to significant remobilization of copper to the dissolved phase. This finding supports the conclusion that the transfer of copper to less bioavailable forms in the sediment is, therefore, effectively irreversible.

## CONCLUSIONS

Available field studies provide a consistent picture of the fate copper that is added to surface water. Studied systems indicate that copper is removed from the water column by a combination of natural processes. Specifically, dissolved copper binds to various functional groups present on SPM in the water column and is transported to sediments via settling. In sediments, further transformations may occur (e.g., through precipitation with sulfide in anoxic systems) as well as burial to deeper sediment layers, resulting in a permanent sequestration of copper. This process represents a transfer of copper to less bioavailable and less toxic forms that is effectively irreversible. Although organic substances can adsorb to settling particles and be transported to the sediment, there is no fundamental change in speciation, as is the case for copper and other metals.

The TICKET–UWM, used as a screening tool, was parameterized to assess the transport and fate of copper in a standardized lake environment. Overall, the outcomes of this modeling were consistent with the available field data. The selection of standardized model parameters was a critical element. However, a sensitivity analysis showed that varying several critical parameters did not affect the main conclusions.

Both field and modeling data were successfully combined in a framework that relates metal removal processes in lentic systems to lake depth and particle settling fluxes. The available literature data indicate that oxidation of anoxic sediments or resuspension events generally do not lead to a significant remobilization of copper, due to the strong binding of copper to various particulate phases. Taken together, the information on the fate of copper in natural waters, including a quantification of removal rates, may be useful in the context of hazard assessment of copper.

## Supplemental Data

The Supplemental Data are available on the Wiley Online Library at DOI: 10.1002/etc.4440.

## Disclaimer

S. Baken and K. Delbeke performed their research as employees of the European Copper Institute.

## Data Accessibility

Data, associated metadata, and calculation tools are available from the corresponding author (krader@mutchassociates.com).

## Supporting information

This article includes online‐only Supplemental Data.

This article includes online‐only Supplemental Data.
