# Supplementary material for: The Fate of Copper Added to Surface Water: Field, Laboratory, and Modeling Studies
Source: Environ Toxicol Chem. 2019 Jun 24;38(7):1386–99. doi: 10.1002/etc.4440 (PMC6852694; doi:10.1002/etc.4440)
Supplement: Supplementary file 1 — This article includes online‐only Supplemental Data. [file ETC-38-1386-s001.pdf]

THE FATE OF COPPER ADDED TO SURFACE WATER: FIELD, LABORATORY, AND MODELING STUDIES

Supporting Information

Kevin J. Rader*[1], Richard F. Carbonaro[1,2], Eric D. van Hullebusch[3], Stijn Baken[4], Katrien Delbeke[4]

[1] Mutch Associates, LLC, Ramsey, New Jersey, USA
[2] Chemical Engineering Department, Manhattan College, Riverdale, New York, USA
[3] Institut de Physique du Globe de Paris, Sorbonne Paris Cité, Université Paris Diderot, Paris, France
[4] European Copper Institute, Brussels, Belgium
* Corresponding author

TRACE METAL REMOBILIZATION REVIEW TABLE

Table SI-1: Metal Remobilization from Studies Discussed in Skeaff et al. (2002)

Source	Water Body	Key Finding as Summarized in (Skeaff et al., 2002)
Diamond et al. (1990)	Enclosures in Perch Lake in Ontario, Canada	Assuming iron and cobalt are representative of first row transition metals, one would expect water-to-sediment rate constants for copper to be in the range of 200 - 300 times the reverse.
Xue et al. (1997)	Lakes Sempach and Greifen in Switzerland	No copper remobilization from Lake Greifen; partial copper release from Lake Sempach likely enhanced by artificial oxygenation. Copper released redeposited with precipitated iron/manganese oxides.
van den Berg et al. (1999)	River Meuse in the Netherlands	Degree of trace metal (including copper) remobilization less than 2% amount deposited.
Hunt and Smith (1983)	Seawater microcosms using sediments from Narragansett Bay, Rhode Island, USA	Observed a release of copper and other metal from contaminated sediments but cautioned that extrapolation to natural systems would require consideration of additional factors.
Zwolsman et al. (1997)	Scheldt estuary in the Netherlands and Belgium.	Dissolved copper and other metals were mobilized in estuary through the deoxidation of sulfides in suspended matter upon encountering the higher oxygen concentrations associated with the lower estuary.
Shaw et al. (1990)	Sites of the coast of California, USA	Metal transport to and from the sediment is complex; sample location showing Cr, Co, Ni, and Cu release had concentrations lower than 2 µg/L
Klinkhammer et al. (1982)	two deep ocean sites in the central equatorial Pacific	Copper flux from sediment to overlying water was 5-10 greater than flux to sediment; however, copper concentrations near the sediment interface were low (0.03 - 0.45 µg/L)

TICKET-UWM SIMULATIONS

BACKGROUND INFORMATION ON THE TICKET-UWM

A Unit World Model (UWM) is a screening level model used to assess the fate and effects of chemicals through simultaneous consideration of chemical partitioning, transport, reactivity, and bioavailability (Mackay, 1979, 1991; Mackay and Paterson, 1991). A UWM for metals has recently been developed that explicitly includes a description of the effect of metal speciation in assessing the long-term fate and effects of metals in the environment (Figure SI-1) (Farley et al., 2007; Farley et al., 2011).

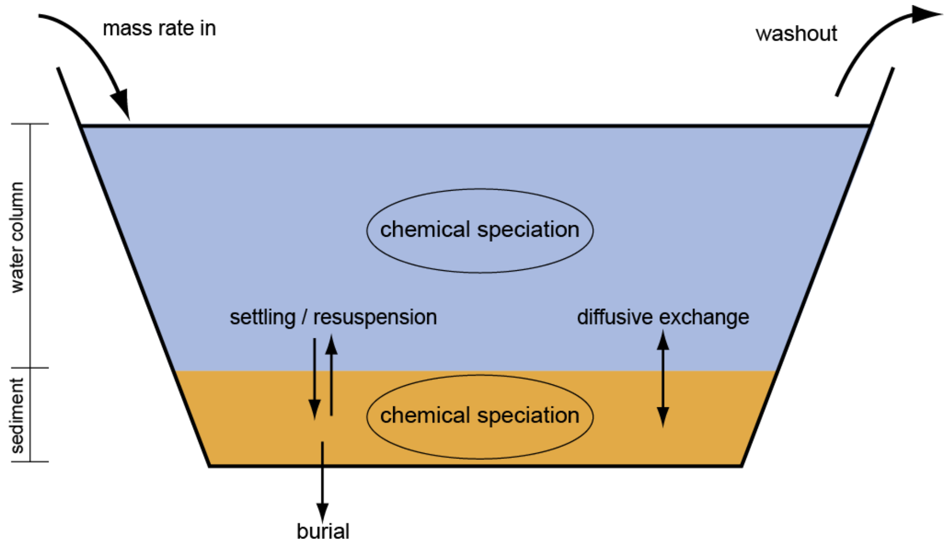

Figure SI-1: Conceptual model for the TICKET Unit World Model for Metals in Lakes (TICKET-UWM).

The model builds on previous screening-level calculations that have been developed for organic contaminants. Unlike previous models, however, the UWM for Metals in Lakes explicitly considers the effects of chemical speciation on metal partitioning, transport and bioavailability in the lake water column and underlying sediments. The numerical engine for the model calculations is the Tableau Input Coupled Kinetics Equilibrium Transport (TICKET) model (Farley et al., 2008). The model domain consists of a single oxic water column layer and a single sediment layer. Specific processes considered in the UWM for

Metals in Lakes (hereafter referred to as the TICKET-UWM) include (i) dissolved and particulate phase transport between the overlying water and sediment; (ii) metal binding to inorganic ligands, dissolved organic carbon (DOC) and particulate organic carbon (POC) (using information from WHAM (Tipping, 1993, 1994; Tipping, 1998; Tipping and Hurley, 1992; Tipping et al., 2011), hydrous ferric oxide (HFO) (Dzombak and Morel, 1990), and hydrous manganese oxide (HMO) (Tonkin et al., 2004); (iii) metal binding to biological receptors using information from the Biotic Ligand Model (BLM) (Di Toro et al., 2001; Santore et al., 2001); (iv) metal precipitation as (hydr)oxides, carbonates, sulfates, and sulfides using information from MINEQL+ software (Schecher and McAvoy, 2003); (v) dissolution kinetics for metal powders, massives, concentrates; average-annual cycling of organic matter and sulfide production in the lake; and (vi) simplified HFO and HMO cycles.

DETAILS OF EUSES MODEL LAKE TICKET-UWM SIMULATIONS

Water Column Simulations

The water chemistries for the EUSES Model Lake TICKET-UWM simulations were based on directives in the EUSES 2.0 report (EC, 2004) and Annex 10 of the *Globally Harmonized System of Classification and Labelling of Chemicals* (GHS) (United Nations, 2017):

- Suspended solids concentrations was specified as 15 mg/L (EC, 2004). Using the this concentration and f_{OC} = 0.1 (EC, 2004), the POC used was 1.5 mg/L.
- Annex 10 reports a TOC limit of 2 mg/L. This annex describes a laboratory protocol for transformation/dissolution experiments in control solutions. Since the scope of these experiments does not include the introduction of POC, the TOC limit above is interpreted as a DOC value. Therefore, the DOC value of the generalized lake environment simulations was set at 2 mg/L.

- The solution composition (major cations/anions) for 3 different pH values was based upon data in Table A10.1 from Annex 10.

Copper addition was modeled in TICKET-UWM simulations as a single instantaneous load of a soluble copper salt (e.g., $CuSO_4 \cdot 5H_2O$). Soluble salts dissolve instantaneously and completely in the water column. This contrasts with metal powers/massives which may dissolve at slower rates, may be only sparingly soluble, and, depending on particle size and density, may be subject to rapid settling. Modeling the addition of copper as a salt, therefore, represents a worst-case scenario for metal release and persistence in the water column.

There exist three potential approaches for assessing copper removal in the model:

1. Copper is added to the model water column as a soluble salt where dissolution is instantaneous (unlike powders or massives which take time to dissolve). The initial copper salt concentration (i.e. nominal or added concentration) is set equal to a relevant value. Based upon the assumption of equilibrium speciation (partitioning), the added copper immediately partitions between the suspended particles and solution phase resulting in an initial dissolved copper concentration less than the initial copper added to the computational system. This initial instantaneous copper removal from the soluble phase to the particles is counted toward the percent removal. After the initial removal, copper removal continues (non-instantaneously) as the finite amount of total copper added to the system maintains its equilibrium with suspended particles and these particles continually settle to the sediment bed. This approach includes two removal components: 1) removal of initially soluble metal associated with the immediate establishment of solid-solution equilibrium and 2) the removal associated with the settling of particles. According to this approach, the fraction remaining is calculated by dividing the dissolved metal concentration at time t, $C_{\mathrm{Diss}}(t)$, by the initial total metal concentration, $C_{\mathrm{Tot}}(0)$. Hereafter this will be referred to as Approach 1.

2. The initial total copper concentration is set equal to a relevant value, and the removal of total copper (particulate + dissolved) in the water column is tracked. According to this approach, the fraction remaining is calculated by dividing the total metal concentration at time t, $C_{Tot}(t)$, by the initial total metal concentration, $C_{Tot}(0)$. This approach is conservative in that it assumes that copper on particles is equally bioavailable as dissolved copper. Hereafter this will be referred to as Approach 2.

3. The initial total copper concentration is set at the relevant value, and the removal of dissolved copper is tracked. According to this approach, removal is calculated by dividing the dissolved copper concentration at time t, $C_{Diss}(t)$, by the initial dissolved copper concentration, $C_{Diss}(0)$. Hereafter this will be referred to as Approach 3.

Approaches 1 and 3 are more consistent with the GHS since they focus on soluble metal. Emphasis is placed on simulation results from Approach 1 but results from Approaches 2 and 3 are indicated as well. If slow enough, the kinetics of the adsorption processes may influence the copper removal rate for Approach 1. However, this analysis is beyond the scope of the current work and is a subject for future investigations. Available data on adsorption rates in natural system indicate considerable variation in the timeframe required for copper adsorption. Estimated equilibration times (i.e., $4[k_f' + k_b]^{-1}$) range from a couple of hours to as much as three weeks (Gee and Bruland, 2002; Herzl et al., 2003; Millward et al., 1992).

The initial total copper concentration (i.e. the spiking concentration) in the water column was the values contained in Table SI-2.

Table SI-2: Summary of Initial Copper Concentrations Used for TICKET-UWM Simulations

Metal	pH 6		pH 7		pH 8	
	Chronic	Acute	Chronic	Acute	Chronic	Acute
Copper	20	25	7	35	11	30

All concentration in units of µg/L

A summary of TICKET-UWM parameters used for the EUSES Model Lake calculations is shown in Table SI-3. The chemical composition associated with the three water chemistries in Table SI-3 is generally consistent with summaries prepared from EU monitoring databases (ARCHE, 2013).

Table SI-3: TICKET-UWM Input for the EUSES Model Lake Simulations

Parameter	Value		
	EUSES Model Lake		
Volume, m^3	3.6×10^9 [a]		
Surface area, km^2	1200 [a]		
Average water depth, m	3 [a]		
Residence time, yr	0.110 [a]		
Settling rate, m/d	2.5 [a]		
Burial rate, cm/yr	0.3 [a]		
Resuspension rate, cm/yr	2.44 [b]		
Diffusive exchange, cm/day	0.24 [a,c]		
Sediment f_{oc}	0.05 [a]		
Sediment solids conc., g/L_{bulk}	500 [a]		
Depth of active sediment, cm	3 [a]		
AVS, $\mu mol/g_{dry}$	0.77 [d]		
Sediment HFO, mg/kg	0 (anoxic sediment)		
Sediment HMO, mg/kg	0 (anoxic sediment)		
Initial Cu conc., µg/L as Cu	35		
Initial Sed Cu conc., µg/g	0		
Suspended solids, mg/L	15 [a]		
Suspended solids foc	0.1 [a]		
POC, mg/L	1.5 [a]		
DOC, mg/L	2.0 [e]		
Water column pH	6.09 [e]	7.07 [e]	8.00 [e]
Sediment pH	6.09	7.07	8.00
Alkalinity, mg/L as $CaCO_3$	3.85	7.47	37.2
Calcium, mg/L	8.0	32.1	80.1
Magnesium, mg/L	1.2	4.9	12.1
Sodium, mg/L	1.8	3.4	18.0
Potassium, mg/L	0.3	1.2	3.02

Table SI-3: TICKET-UWM Input for the EUSES Model Lake Simulations

Parameter	Value		
	EUSES Model Lake		
Sulfate, mg/L as SO_4	4.8	19.2	47.9
Chloride	14.5	57.8	145

[a] Data from EUSES model lake (EC, 2004; European Chemicals Agency, 2010). Surface area and volume values quoted refer to a regional scale assessment. They do not influence the removal simulations.
[b] Calculated using the settling velocity, suspended solids concentration, sediment bulk solid concentration, and the burial (net sedimentation) rate shown in the table using a steady-state solids balance (Chapra, 1997).
[c] EUSES pore water side mass transfer coefficient. Mass transfer resistance is assumed to be all on the sediment side (Di Toro et al., 1981).
[d] 10th percentile AVS concentration for a Flemish dataset (Vangheluwe et al., 2005)
[e] Water chemistries for the three pH values were taken from Annex 10 of the Globally Harmonized System of Classification and Labelling of Chemicals (United Nations, 2017).

The resuspension rate (Table SI-3) was calculated from a solid mass balance in the active sediment layer assuming steady-state conditions (no accumulation/depletion of sediment solids). The following equation was used:

$$v_r = v_s \frac{m}{m_a} - v_b \qquad \text{(SI-1)}$$

where v_s is the settling velocity (2.5 m/d), m is the water column suspended solids concentration (15 mg/L), m_a is the sediment solids concentration (0.5 kg/L), and v_b is the burial rate (0.3 cm/yr). To be consistent with laboratory/mesocosm/field removal tests, the diluting effect of flow into the system was minimized by increasing the hydraulic residence time of the model lake system from the EUSES value of 40 days (0.11 year) to 300 years.

It should be noted that the EUSES Model Lake is prescribed to have a sediment compartment that is 10% aerobic. At present, TICKET-UWM simulations utilize a single sediment layer and cannot directly reproduce this condition. The redox state (oxic or anoxic) of the sediment layer in the TICKET-UWM is specified by the user. This selection determines the sorption/precipitation reactions included in the simulation. For the EUSES Model Lake calculations, an anoxic sediment layer was used with the AVS

concentration set to the 10th percentile value from EU monitoring data (Vangheluwe et al., 2005) (additional information from: http://echa.europa.eu/copper-voluntary-risk-assessment-reports [environment/Risk Characterization/Chapter 3.3.7.1.3.]). This reflects a level of conservatism with respect to the amount of copper capable of being bound by sulfide in sediments.

Simulations were made using empirical K_D values and K_D values calculated by WHAM VII (Tipping et al., 2011). For the Empirical K_D simulation, the surface water and sediment log K_D values were specified as 4.48 and 4.39 based on the copper partition coefficient review of Heijerick and Van Sprang (2005). For the Calculated K_D simulation, K_D values at each time step were generated with WHAM VII. Copper binding to HFO and HMO in the water column was not considered.

Based on the suspended solids concentration of 15 mg/L and log K_D of 4.48, approximately 31% of the copper added to the water column was bound to suspended particles for the Empirical K_D simulation for the EUSES Model Lake. The decrease of copper in time for the Empirical K_D and Calculated K_D simulations is shown below in Figure SI-2. Summaries of EUSES Model Lake TICKET-UWM simulation results are presented in Table SI-4 through Table SI-7 below.

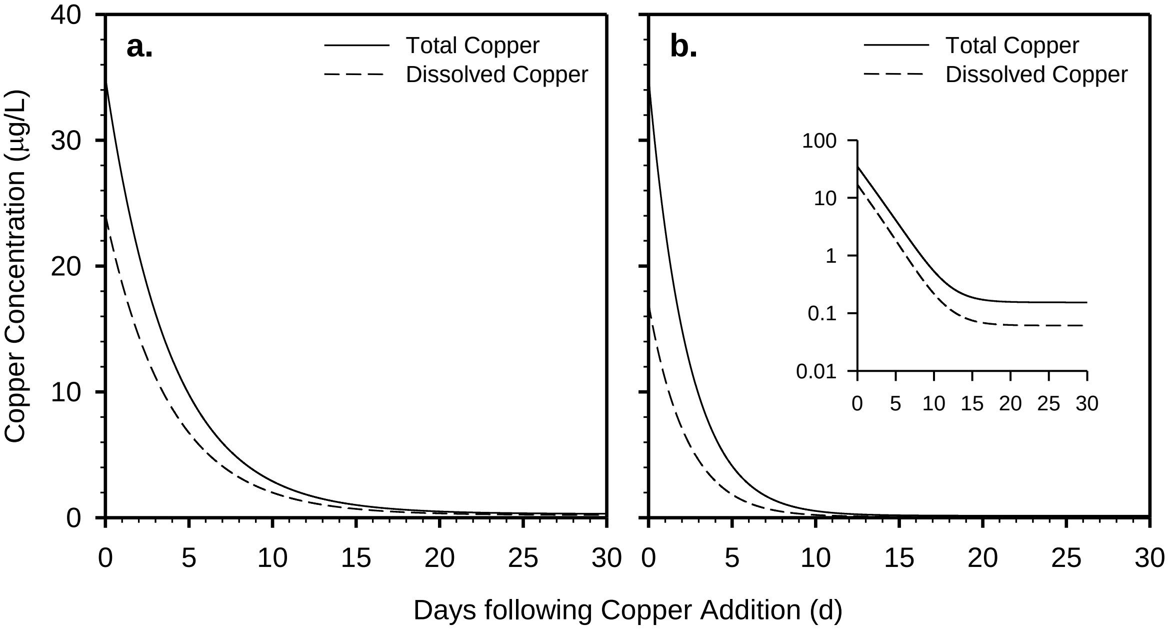

Figure SI-2: Time series of total and dissolved copper for the EUSES Model Lake with K_D a) specified at empirical values (surface water log K_D: 4.48; sediment log K_D: 4.39) and b) calculated at each time step using a WHAM VII speciation calculation.

Table SI-4: Summary of EUSES Model Lake Empirical K_D Simulation Results

Removal Approach and Output Quantity	Result from Model
**1: $C_D(t)/C_T(0)$**	
Time for 70% removal, days	3.25
log K_D range [b]	4.48
Fraction particulate range [b]	0.312
**2: $C_T(t)/C_T(0)$**	
Time for 70% removal, days	4.72
log K_D range [b]	4.48
Fraction particulate range [b]	0.312
**3: $C_D(t)/C_D(0)$**	
Time for 70% removal, days	4.72
log K_D range [b]	4.48
Fraction particulate range [b]	0.312

[a] 70% removal occurs instantly via initial solid-solution equilibrium partitioning. Instead of log K_D and fraction particulate value ranges, the values at t = 0 are indicated.

[b] These ranges were calculated using data prior to the time at which 70% removal was achieved.

Table SI-5: Summary of EUSES Model Lake Calculated K_D Simulation Results for Different Water Chemistries and Copper Loadings

Removal Approach and Output Quantity	pH = 6.09		pH = 7.07		pH = 8.00	
	20 µg/L	25 µg/L	7 µg/L	35 µg/L	11 µg/L	30 µg/L
**1: $C_D(t)/C_T(0)$**						
Time for 70% removal, days	0.922	0.961	0.918	1.11	1.01	1.34
Log K_D range [a]	4.89 - 4.91	4.87 - 4.90	4.89 - 4.91	4.85 - 4.86	4.85 - 4.89	4.77 - 4.81
Fraction particulate range [a]	0.537 - 0.548	0.529 - 0.542	0.539 - 0.549	0.513 - 0.521	0.514 - 0.538	0.470 - 0.492
**2: $C_T(t)/C_T(0)$**						
Time for 70% removal, days	2.69	2.71	2.68	2.83	2.72	2.98
Log K_D range [a]	4.89 - 4.93	4.87 - 4.93	4.89 - 4.94	4.85 - 4.88	4.85 - 4.94	4.77 - 4.86
Fraction particulate range [a]	0.537 - 0.561	0.529 - 0.559	0.539 - 0.568	0.513 - 0.531	0.514 - 0.567	0.470 - 0.523
**3: $C_D(t)/C_D(0)$**						
Time for 70% removal, days	2.57	2.57	2.54	2.75	2.48	2.75
Log K_D range [a]	4.89 - 4.93	4.87 - 4.92	4.89 - 4.94	4.85 - 4.88	4.85 - 4.94	4.77 - 4.86
Fraction particulate range [a]	0.537 - 0.560	0.529 - 0.558	0.539 - 0.567	0.513 - 0.530	0.514 - 0.564	0.470 - 0.518

[a] These ranges were calculated using data prior to the time at which 70% removal was achieved.

Table SI-5. Continued

Removal Approach and Output Quantity	pH = 6.09		pH = 7.07		pH = 8.00	
	100 µg/L	1,000 µg/L	100 µg/L	1,000 µg/L	100 µg/L	1,000 µg/L
**1: $C_D(t)/C_T(0)$**						
Time for 70% removal, days	1.37	4.16	1.35	4.37	1.99	--- a
log K_D range [a]	4.75 - 4.80	4.21 - 4.46	4.75 - 4.81	4.18 - 4.45	4.58 - 4.69	5.82
Fraction particulate range [a]	0.458 - 0.489	0.194 - 0.303	0.459 - 0.492	0.186 - 0.295	0.361 - 0.425	0.908
**2: $C_T(t)/C_T(0)$**						
Time for 70% removal, days	3.00	5.52	3.00	5.72	3.51	1.85
log K_D range [a]	4.75 - 4.86	4.21 - 4.56	4.75 - 4.85	4.18 - 4.55	4.58 - 4.77	5.19 - 5.82
Fraction particulate range [a]	0.458 - 0.522	0.194 - 0.353	0.459 - 0.516	0.186 - 0.345	0.361 - 0.469	0.701 - 0.908
**3: $C_D(t)/C_D(0)$**						
Time for 70% removal, days	2.73	4.85	2.74	5.05	3.07	8.86
Log K_D range [a]	4.75 - 4.85	4.21 - 4.51	4.75 - 4.85	4.18 - 4.50	4.58 - 4.75	4.42 - 5.82
Fraction particulate range [a]	0.458 - 0.517	0.194 - 0.327	0.459 - 0.514	0.186 - 0.320	0.361 - 0.458	0.281 - 0.908

[a] These ranges were calculated using data prior to the time at which 70% removal was achieved.

Table SI-6: Summary of EUSES Model Lake Simulation Results for Settling Velocity of 0.24 m/d

Metal Removal Approach and Output Quantity	Empirical KD	Calculated K_D		
		pH = 6.09	pH = 7.07	pH = 8.00
**1: $C_D(t)/C_T(0)$**				
Time for 70% removal, days	32.6	9.73	11.2	13.5
log K_D range [a]	4.48	4.87 - 4.90	4.85 - 4.86	4.77 - 4.81
Fraction particulate range [a]	0.312	0.529 - 0.543	0.513 - 0.521	0.470 - 0.492
**2: $C_T(t)/C_T(0)$**				
Time for 70% removal, days	47.3	27.4	28.6	30.1
log K_D range [a]	4.48 - 4.48	4.87 - 4.93	4.85 - 4.88	4.77 - 4.87
Fraction particulate range [a]	0.312 - 0.312	0.529 - 0.559	0.513 - 0.531	0.470 - 0.525
**3: $C_D(t)/C_D(0)$**				
Time for 70% removal, days	47.3	26.0	27.7	27.8
log K_D range [a]	4.48 - 4.48	4.87 - 4.93	4.85 - 4.88	4.77 - 4.86
Fraction particulate range [a]	0.312 - 0.312	0.529 - 0.558	0.513 - 0.531	0.470 - 0.519

[a] These ranges were calculated using data prior to the time at which 70% removal was achieved.

Table SI-7: Summary of EUSES Model Simulation Results with DOC increased from 2 to 15 mg/L

Metal Removal Approach and Output Quantity	Calculated K_D		
	**pH = 6.09**	**pH = 7.07**	**pH = 8.00**
**1: $C_D(t)/C_T(0)$**			
Time for 70% removal, days	8.43	9.08	8.67
log K_D range [a]	4.07 - 4.08	4.02 - 4.06	4.02 - 4.08
Fraction particulate range [a]	0.149 - 0.151	0.135 - 0.146	0.137 - 0.152
**2: $C_T(t)/C_T(0)$**			
Time for 70% removal, days	9.79	10.4	10.0
log K_D range [a]	4.06 - 4.08	4.02 - 4.06	4.02 - 4.08
Fraction particulate range [a]	0.148 - 0.151	0.135 - 0.148	0.137 - 0.152
**3: $C_D(t)/C_D(0)$**			
Time for 70% removal, days	9.82	10.3	9.87
log K_D range [a]	4.06 - 4.08	4.02 - 4.06	4.02 - 4.08
Fraction particulate range [a]	0.148 - 0.151	0.135 - 0.148	0.137 - 0.152

[a] These ranges were calculated using data prior to the time at which 70% removal was achieved.

Detailed Sediment Simulations

The water column in the TICKET-UWM is represented as being oxic with a negligible sulfide concentration. In accordance with the equilibrium mass action law, any copper sulfide solid resuspended from the sediment layer to the water column immediately dissolves, releasing copper to re-equilibrate between the settling particles the dissolved phase in the water column. Since the immediate redistribution supplies dissolved copper in the water column, the model calculations are closer to a worst-case scenario for dissolved copper removal.

For the detailed sediment simulations, the EUSES Model Lake water chemistry (Table SI-3) was used for the surface water. Model simulations used bulk and pore water sediment chemistry from several field studies (Table SI-8). The base case sediment pH was 7.56. Copper log K_D values in sediment were specified using the Calculated K_D approach (described above). Simulations were performed for an oxic sediment and an anoxic sediment to assess the two redox end members occurring in natural sediment. For the oxic sediment simulations, sulfide production and copper sulfide precipitation were not included. Copper was allowed to sorb to POC, HFO, and HMO in the sediment and precipitate as carbonates and/or hydroxides. For the anoxic sediment simulations, copper binding to HFO and HMO was not considered. Copper was allowed to sorb to POC and precipitate as sulfides, carbonates, and/or hydroxides.

To model the formation and dissolution of copper sulfide solid, the following reaction and solubility product from Dyrssen and Kremling (1990) were used initially:

$$CuS(s) + H^+ = Cu^{2+} + HS^- \qquad \log {}^*K_s = -22.3 \qquad \text{(SI-2)}$$

Simpson et al. (2000) suggest Cu_2S is an important copper solid phase in anoxic sediments. The solubility of this species has been described with the following solubility product in (Dyrssen and Kremling, 1990):

$$Cu_2S(s) + H^+ = 2Cu^+ + HS^- \qquad \log {}^*K_s = -34.65 \qquad \text{(SI-3)}$$

Simulations were made with both copper-sulfide solid species.

Table SI-8: Bulk and Pore Water Sediment Chemistry Parameters

Parameter	Value
pH	7.56 [a]
Ca^{2+}	144 [a]
Mg^{2+}	38.1 [a]
Na^{+}, mg/L	141 [a]
K^{+}, mg/L	6.19 [a]
Cl^{-}, mg/L	79 [a]
SO_4^{2-}, mg/L	65 [a]
Alkalinity, mg/L as $CaCO_3$	478 [b]
DOC, mg/L	21 [c]
TOC, %	3.7 [d]
Iron, %	2.9 [e]
HFO, mg HFO/kg	18,600 [f]
Manganese, mg/kg	404 [g]
HMO, mg HMO/kg	154 [f]
AVS, μmol/g $_{dry}$	1, 9.1 [h]
Pore water [Fe^{2+}], mg/L	0.912 [i]
Settling rate, m/d	2.5 [j]
Burial rate, cm/yr	0.3 [j]
Resuspension rate, cm/yr	2.44 [j]
Diffusive exchange, cm/day	0.24 [j]
Sediment solids conc., g/L	500 [j]
Active depth, cm	3 [j]

[a] From a monitoring dataset on Flemish navigable waterways (de Deckere et al., 2000). For conversion of hardness to Ca^{2+} and Mg^{2+}, a molar ratio of Ca:Mg = 2.20 was used based on average river water in Stumm and Morgan (1996). Na^{+} and K^{+} were estimated using the SO_4^{2-} and Cl^{-} data from (de Deckere et al., 2000) and a seawater/river water mixing method similar to Di Toro et al. (2005).

[b] Unpublished data from Besser et al. (2011).

[c] 50th percentile of data from Flemish sediments **(AMINAL/AWZ, 1995)**.

[d] Geometric mean of 10th percentile values from 4 datasets: Netherlands (0.001; 0.029, 0.034, 0.045, 0.052 and 0.07 g organic carbon/g dry wt; M. Vangheluwe, personal communication), Finland, UK, and Spain **(EURAS, 2008)**.

[e] Geometric mean of 50th percentile values from 3 datasets: Finland, UK, and Spain **(EURAS, 2008)**.

[f] Determined using a relationship between total recoverable Fe/Mn and the Fe/Mn in a sequential extraction fraction most closely related to HFO/HMO as determined by HydroQual and Manhattan College (2010) using the following formula weights: 89 g HFO/mol Fe and 119 g HMO/mol Mn.

[g] Besser et al. (2011).

[h] Geometric mean of 10th and 50th percentile values from 5 datasets: Flanders and Netherlands **(Vangheluwe et al., 2005)**, Finland, UK, and Spain **(EURAS, 2008)**.

[i] An average dissolved Fe(II) in the porewater of 2.77 mg/L was calculated based on data from Wersin et al. (1991), Gallon et al. (2004), and Canavan et al. (2007). Using the porewater chemistry data in this table, WHAM6 calculations were used to determine the ferrous ion concentration, [Fe^{2+}].

[j] Values from Table SI-3**Table SI-12**

Water column results for the anoxic simulation indicate that around day 24 of the simulation, the total and dissolved copper concentrations leveled off. Similar behavior was noted for the water column runs (Figure SI-2). For the remainder of the 365-day simulation, water column copper concentrations decreased only very slightly. Between day 24 and day 365, the ranges of water column copper concentrations were 140 to 160 ng/L (total) and 56 to 62 ng/L (dissolved) respectively. These dissolved values are greater than 150 times lower than the 70% removal concentration of 10.5 μg/L. The relatively flat copper response between day 24 and 365 in the water column was the result of a local equilibrium established between the settling flux of copper (directed into the sediment) and the resuspension flux (directed out of the sediment). The local equilibrium can be referred to as a *pseudo steady-state*. The smaller burial flux slowly depleted copper from the system moving the water column / sediment system toward the final steady-state in which the water column and sediment copper concentrations were zero. Detailed output from anoxic and oxic simulations is shown in Table SI-9.

Table SI-9: Results of Sediment Simulations

**Sediment Type**		**Anoxic**		**Anoxic (using Cu_2S)**	**Oxic**
**AVS, μmol/g**		**9.1**	**1**	**9.1**	**0**
Water Column Removal Data					
Time for 70% Removal	days	3.4	3.4	3.4	3.4
$[0.3\times C_T(0)]$/Max QSS C_T [i]		67	67	68	66
Pseudo Steady-State Water Column Copper Concentrations					
Tot. Cu Range [a]	ng/L	140 - 160	140 - 160	140 - 150	140 - 160
Diss. Cu Range [a]	ng/L	56 - 62	56 - 62	56 - 61	57 - 64
Water column log K_D [a]	--- [b]	5.00	5.00	5.00	5.00
Mass Balance at 1 Year					
Mass in the Water Column	%	0.40	0.40	0.40	0.41
Mass in the Sediment	%	90.2	90.2	90	90
Total Settling IN	%	176	176	176	178
Total Resusp. OUT	%	76.6	76.6	77	76.6
Total Burial OUT	%	9.42	9.42	9.4	9.41
Total Diffusion NET [c]	%	0.133	0.133	0.133	-1.47
Sediment Speciation Data [d]					
Total Sediment Cu	μg/g	6.9	6.9	6.9	6.9
	μmol/g	0.11	0.11	0.11	0.11
"Free" Cu^{2+}	ng/L_{pw} [e]	1.5E-13	1.5E-13	1.7E-14	3.1E-01

Table SI-9: Results of Sediment Simulations					
Sediment Type		Anoxic		Anoxic (using Cu_2S)	Oxic
AVS, µmol/g		9.1	1	9.1	0
	µg/g	2.4E-19	2.4E-19	2.6E-20	5.0E-07
Inorganic Cu [f]	ng/L_{pw}	2.7E-12	2.7E-12	2.1E-07	4.2E+00
	µg/g	4.4E-18	4.4E-18	3.4E-13	6.7E-06
Cu-DOC	ng/L_{pw}	7.0E-10	7.0E-10	7.8E-11	2.0E+03
	µg/g	1.1E-15	1.1E-15	1.2E-16	3.2E-03
Cu-POC	µg/g	1.7E-12	1.7E-12	1.9E-13	6.6E+00
Cu-HO&C [g]	µg/g	0	0	0	0
Cu-Sulfide	µg/g	6.9	6.9	6.9	0
Cu-HFO	µg/g	9.9E-23	9.9E-23	1.1E-23	3.0E-01
Cu-HMO	µg/g	6.8E-24	6.8E-24	7.5E-25	2.5E-02
Dissolved Cu	ng/L_{pw}	7.1E-10	7.1E-10	2.1E-07	2.0E+03
	µg/g	1.1E-15	1.1E-15	3.4E-13	3.2E-03
Particulate Cu	µg/g	6.93	6.93	6.93	6.93
Sediment log K_D	--- [h]	16.0	16.0	13.5	3.53

a These refer to the period in the simulation from day 24 to day 365. The log K_D values quoted are arithmetic averages for this time period

b Units of water column K_D are L/kg

c This number is the diffusive flux integrated over the entire simulation time. Negative diffusive flux values are *directed out of the sediment* and positive diffusive flux values are directed *into the sediment*.

d Speciation data was taken from day 20 of the simulation

e pw = porewater

f Inorganic copper is the sum of the inorganic copper complexes (e.g., with hydroxide, chloride and carbonate)

g Copper (hydr)oxide and copper carbonate precipitates

h Units of sediment K_D are L_{pw}/kg

i This quantity is the ratio of the total Cu concentration representing 70% removal ($0.3 \times C_T(0)$) to the maximum total concentration during the quasi-steady-state period (Max QSS C_T). This is meant to give an indication of where sustained water column concentrations lie relative to the 70% removal benchmark.

A series of sensitivity analyses were conducted to assess effect of (i) low AVS (1 µmol/g versus the base case value of 9.1 µmol/g), (ii) variation in water column/sediment pH (6/7, 7/7, and 8/7.5 versus the base case combination of 7.07/7.56), (iii) low sediment solids concentration (150 g/L_{bulk} versus the base case value of 500 g/L_{bulk}), (iv) variation in hardness (factor of 2 about the base value of 516 mg/L as $CaCO_3$), (v) variation in resuspension rate (0.1, 1, 3.2, and 10 times the default rate of 2.44 cm/yr), and (vi) variation in copper loading (initial copper concentrations of 10, 100, and 1000 µg/L).

Model results from the anoxic simulation with AVS of 1 µmol/g were identical to those with an AVS of 9.1 (Table SI-9). This is because AVS was present in excess of the total sediment copper concentration.

Simulations associated with items (ii) - (iv) use an anoxic simulation with $Cu_2S(s)$ and total initial copper of 100 µg/L. Detailed results can be found in Table SI-10. For the relative pH variation sensitivity analysis, the largest change in time required to achieve 70% removal of total copper was for the 8/7.5 simulation; the predicted increase in removal time was from 3.5 days to 3.9 days. Variation of pH water column / sediment pH values had a relatively minor impact on the magnitude of the pseudo steady-state water column copper concentrations (Table SI-10). Total copper concentrations at the start of pseudo steady state were within 10% of the base case. Dissolved concentrations from the pH 8/7.5 simulation were slightly higher than the base case (i.e., 7.07/7.56). However, the total copper concentrations at the start of pseudo state-state conditions were at least 60 times smaller than the concentration representing 70% removal. The mass balance results were similar to the base case. In all pH variation runs, particulate copper speciation in the sediment was dominated by the formation of $Cu_2S(s)$. Removal time, pseudo steady-state water column copper concentrations, and mass balance results showed no sensitivity to a factor of 2 variation in sediment hardness. Though the simulations with the decreased sediment solid concentration showed some departures from the base case values for pseudo steady-state water column copper concentrations as well as settling and resuspension fluxes, key outcomes remain similar to the base case. The 70% removal time was still around 3.5 days, the total copper concentrations at the start of pseudo state-state conditions was still significantly smaller than the concentration representing 70% removal, the integrated diffusive flux was directed into the sediment and particulate copper speciation in the sediment was dominated by the formation of $Cu_2S(s)$.

Simulations associated with item (v) used an anoxic simulation with AVS = 9.1 µmol/g, CuS(s), and total initial copper of 35 µg/L. Model runs with the sediment resuspension rate set at 0.1, 1, 3.2, and 10 times the default rate (item v) were made to examine the impact of resuspension on water column copper concentrations in more detail. The resuspension rate was multiplied by factors of 0.1, 3.2, and 10 keeping the settling velocity and burial rate constant. The total and dissolved copper concentration in the water column at day 15 increased as the resuspension rate increased (Figure SI-3). However, even at the highest

resuspension rate (i.e., 10 times the default value), total and dissolved copper in the water remained more than 6 times lower than the concentration representing 70% removal of 10.5 µg/L.

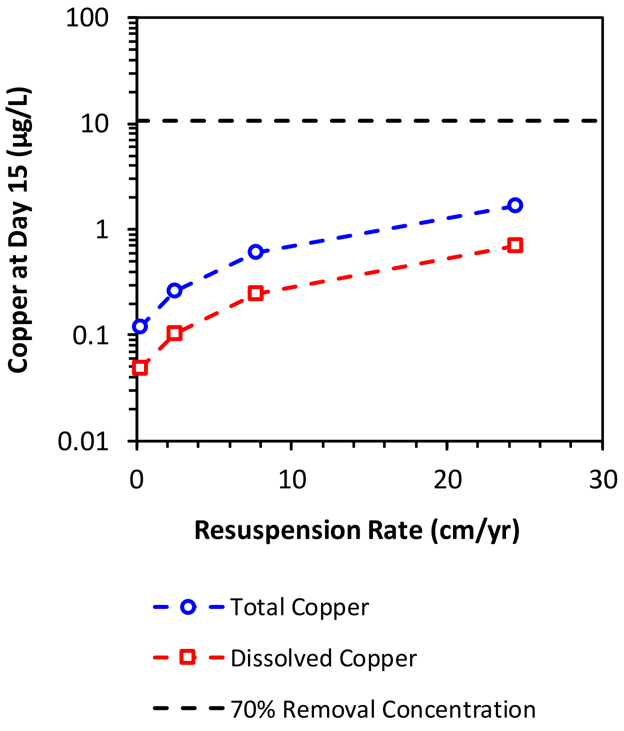

Figure SI-3: Effect of resuspension rate on total and dissolved copper concentration at day 15.

Simulations associated with items (vi) used an anoxic simulation with AVS = 9.1 µmol/g and CuS(s). The results indicate that total dissolved pseudo steady-state copper concentration and mass flux/balance values (except the diffusive flux) varied linearly with the initial copper concentration (Table SI-11). For the sensitivity analysis simulation with initial copper concentration at 1,000 µg/L and an AVS of 1 µmol/g, the capacity of the sediment to bind copper as CuS(s) was exceeded. Sediment copper in excess of AVS was bound by POC. This results in a decreased sediment K_D, increased pore water copper concentration and an integrated diffusive flux directed out of the sediment. However, this change in sediment speciation does not impact the amount of time required for 70% removal in the water column nor does it influence the pseudo steady-state concentration to an appreciable extent (compare last two columns in Table SI-11). The

copper loading sensitivity analysis simulations show 70% removal times that are less than 6 days and pseudo steady state water column total copper concentrations that are more than 50 times lower than that representing 70% removal.

Empirical K_D sediment simulations indicate total and dissolved copper concentrations at the onset of pseudo steady-state conditions of 0.31 and 0.21 μg/L, respectively, which are more than 30 times lower than the 70% removal concentration of 10.5 μg/L. Although the water column log K_D was greater than the sediment log K_D, the integrated diffusive flux was directed into the sediment.

Table SI-10: Results of Copper Sediment Sensitivity Analysis Runs

Removal Approach and Output Quantity	Sensitivity Analysis Run						
	**Base Case [a]**	**WC pH = 6.094 Sed pH = 7**	**WC pH = 7.073 Sed pH = 7**	**WC pH = 8.002 Sed pH = 7.5**	**Hardness × 2**	**Hardness ÷ 2**	**Sediment solids = 150 g/L_{bulk}**
Tot. Cu Range, µg/L [b]	0.400 – 0.450	0.440 – 0.490	0.410 – 0.450	0.420 – 0.470	0.400 – 0.450	0.400 – 0.450	1.50 – 1.70
Diss. Cu Range, µg/L [b]	0.160 – 0.180	0.200 – 0.220	0.160 – 0.180	0.180 – 0.200	0.160 – 0.180	0.160 – 0.180	0.630 – 0.710
Mass in the Water Column, %	0.40	0.44	0.41	0.42	0.40	0.40	1.50
Mass in the Sediment, %	90.2	90.1	90.2	90.2	90.2	90.2	89.2
Total Settling IN, %	176	176	176	176	176	176	372
Total Resusp. OUT, %	76.6	76.6	76.6	76.5	76.6	76.6	273.7
Total Diffusion NET, % [c]	0.143	0.152	0.143	0.174	0.143	0.143	0.287
Total Burial OUT, %	9.42	9.41	9.42	9.41	9.42	9.42	9.31
Water column log K_D, L/kg [b]	4.99	4.91	4.99	4.94	4.99	4.99	4.95
Sediment log K_D, L/kg [b]	14.0	14.0	14.0	14.0	14.0	14.0	14.5
Time for 70% Removal of Total Copper, days	3.5	3.5	3.5	3.9	3.5	3.5	3.6
$[0.3 \times C_T(0)]$/Max QSS C_T [d]	66	62	66	63	66	66	18

[a] Base-case simulation parameters: water column pH 7.07; sediment pH 7.56; anoxic sediment with AVS = 1 µmol/g, settling velocity 2.5 m/d; initial Cu concentration = 0.1 mg/L; Cu_2S is the potential copper sulfide precipitate

[b] Ranges and average are based on data from the quasi-steady state period of the simulation.

[c] This number is the diffusive flux integrated over the *entire* 365-day simulation. Negative diffusive flux values are directed out of the sediment and positive diffusive flux values are directed into the sediment.

[d] This quantity is the ratio of the total Cu concentration representing 70% removal ($0.3 \times C_T(0)$) to the maximum total concentration during the quasi-steady-state period (Max QSS C_T). This is meant to give an indication of where sustained water column concentrations lie relative to the 70% removal benchmark.

**Table SI-11: Results of Copper Loading Sediment Sensitivity Analysis Simulations**							
**Initial Total Copper, µg/L**		**10**		**100**		**1000**	
**AVS, µmol/g**		**9.1**	**1**	**9.1**	**1**	**9.1**	**1**
Water Column Removal Data							
Time for 70% Removal	days	3.2	3.2	3.5	3.5	6.0	6.0
[0.3×C_T(0)]/Max QSS C_T [i]		68	68	66	66	61	60
Pseudo Steady-State Water Column Copper Concentrations							
Tot. Cu Range [a]	ng/L	40 - 44	40 - 44	400 - 450	400 - 450	4,400 – 4,900	4,500 - 5000
Diss. Cu Range [a]	ng/L	16 - 18	16 - 18	160 - 180	160 - 180	2,000 – 2,200	2,000 – 2,300
Water column log K_D [a]	--- [b]	5.01	5.01	4.99	4.99	4.91	4.91
Mass Balance at 1 Year							
Mass in the Water Column	%	0.40	0.40	0.40	0.40	0.44	0.45
Mass in the Sediment	%	90.2	90.2	90.2	90.2	90.2	90.2
Total Settling IN	%	176	176	176	176	175	177
Total Resusp. OUT	%	76.6	76.6	76.6	76.6	76.2	76.2
Total Burial OUT	%	9.42	9.42	9.42	9.42	9.37	9.36
Total Diffusion NET [c]	%	0.124	0.124	0.143	0.143	0.297	-1.151
Sediment Speciation Data [d]							
Total Sediment Cu	µg/g	2.0	2.0	20	20	198	198
	µmol/g	0.03	0.03	0.31	0.31	3.1	3.1
"Free" Cu^{2+}	ng/L_{pw} [e]	1.5E-13	1.5E-13	1.5E-13	1.5E-13	1.5E-13	6.1E+01
	µg/g	2.4E-19	2.4E-19	2.4E-19	2.4E-19	2.4E-19	9.8E-05
Inorganic Cu [f]	ng/L_{pw}	2.7E-12	2.7E-12	2.7E-12	2.7E-12	2.7E-12	1.1E+03
	µg/g	4.4E-18	4.4E-18	4.4E-18	4.4E-18	4.4E-18	1.8E-03
Cu-DOC	ng/L_{pw}	7.0E-10	7.0E-10	7.0E-10	7.0E-10	7.0E-10	5.3E+04
	µg/g	1.1E-15	1.1E-15	1.1E-15	1.1E-15	1.1E-15	8.5E-02
Cu-POC	µg/g	1.7E-12	1.7E-12	1.7E-12	1.7E-12	1.7E-12	1.3E+02
Cu-HO&C [g]	µg/g	0	0	0	0	0	0
Cu-Sulfide	µg/g	2.0	2.0	19.8	19.8	197.7	63.5
Cu-HFO	µg/g	9.9E-23	9.9E-23	9.9E-23	9.9E-23	9.9E-23	3.8E-08
Cu-HMO	µg/g	6.8E-24	6.8E-24	6.8E-24	6.8E-24	6.8E-24	1.6E-09
Dissolved Cu	ng/L_{pw}	7.1E-10	7.1E-10	7.1E-10	7.1E-10	7.1E-10	5.5E+04
	µg/g	1.1E-15	1.1E-15	1.1E-15	1.1E-15	1.1E-15	8.7E-02
Particulate Cu	µg/g	2.0	2.0	20	20	198	198
Sediment log K_D	--- [h]	15.4	15.4	16.4	16.4	17.4	3.56

[a] These refer to the period in the simulation from day 24 to day 365. The log K_D values quoted are arithmetic averages for this time period

[b] Units of water column K_D are L/kg

[c] This number is the diffusive flux integrated over the entire simulation time. Negative diffusive flux values are *directed out of the sediment* and positive diffusive flux values are directed *into the sediment*.

[d] Speciation data was taken from day 20 of the simulation

[e] pw = pore water

[f] Inorganic copper is the sum of the inorganic copper complexes (e.g., with hydroxide, chloride and carbonate)

[g] Copper (hydr)oxide and copper carbonate precipitates

[h] Units of sediment K_D are L_{pw}/kg

[i] This quantity is the ratio of the total Cu concentration representing 70% removal ($0.3 \times C_T(0)$) to the maximum total concentration during the quasi-steady-state period (Max QSS C_T). This is meant to give an indication of where sustained water column concentrations lie relative to the 70% removal benchmark.

DETAILS OF SAINT GERMAIN LES BELLES RESERVOIR TICKET-UWM SIMULATIONS

Parameter Selection

Where possible, physical and chemical parameters serving as input for the TICKET-UWM were specified based on direct measurements provided in (van Hullebusch et al., 2003a; van Hullebusch et al., 2003b) (Table SI-12). Since settling rate, burial rate, and sediment solids concentration were not measured for the reservoir, these parameters were set to regional values from the EUSES model lake (EC, 2004; European Chemicals Agency, 2010) (Table SI-12). The resuspension rate was calculated based on a solids balance and assuming the solids in the system are at steady-state.

Table SI-12: TICKET-UWM Input for the Saint Germain les Belles Reservoir Simulations[a]

Parameter	Value
	**Saint Germain les Belles Reservoir [a]**
Volume, m^3	6.5×10^4
Surface area, km^2	0.04
Average water depth, m	1.6
Residence time, yr	0.25
Settling rate, m/d	Calibrated
Burial rate, cm/yr	0.3 [b]
Resuspension rate, cm/yr	2.75 [c]
Diffusive exchange, cm/day	0.24 [b,d]
Sediment f_{oc}	0.073
Sediment solids conc., g/L_{bulk}	500 [b]
Depth of active sediment, cm	5
AVS, $\mu mol/g_{dry}$	8.8 [e]
Sediment HFO, mg/kg	0 (anoxic sediment)
Sediment HMO, mg/kg	0 (anoxic sediment)
Initial Cu conc., µg/L as Cu	197 [f]
Initial Sed Cu conc., µg/g	37.7
Suspended solids, mg/L	18.0
Suspended solids foc	0.1 [b]
POC, mg/L	1.80
DOC, mg/L	5.1
Water column pH	8.4
Sediment pH	7.5
Alkalinity, mg/L as $CaCO_3$	49.0
Calcium, mg/L	3.4
Magnesium, mg/L	3.6
Sodium, mg/L	5.1
Potassium, mg/L	1.3
Sulfate, mg/L as SO_4	4.7
Chloride, mg/L	7.5

[a] Unless otherwise noted, input parameter are taken (or estimated) from (van Hullebusch et al., 2003a; van Hullebusch et al., 2003b)

[b] Data from EUSES model lake (EC, 2004; European Chemicals Agency, 2010). Surface area and volume values quoted refer to a regional scale assessment. They do not influence the removal simulations.

[c] Calculated using the settling velocity, suspended solids concentration, sediment bulk solid concentration, and the burial (net sedimentation) rate shown in the table using a steady-state solids balance (Chapra, 1997).

[d] EUSES pore water side mass transfer coefficient. Mass transfer resistance is assumed to be all on the sediment side (Di Toro et al., 1981).

[e] 50th percentile AVS concentration for a Flemish dataset (Vangheluwe et al., 2005)

[f] This was calculated by adding the theoretical dose value to the measured pre-dose background copper concentration of 1.28 µg/L.

Distribution Coefficients - Empirical K_D

According to measured total and dissolved copper and suspended particulate matter (SPM) concentrations in the reservoir, log K_D values spanned the range 3.66 to 4.87 (i.e., $K_D = 10^{3.66}$ to $10^{4.87}$ L/kg). The Empirical K_D simulation used the average log K_D value of 4.56. Based on the average SPM concentration over the study period of 18.0 mg/L, the average fraction particulate was 0.39. The distribution coefficient in the sediment of the reservoir was not measured. Therefore, for the Empirical K_D simulation the sediment log K_D was set at 4.39 based upon the copper partition coefficient review of Heijerick and Van Sprang (2005).

Distribution Coefficients - Calculated K_D

WHAM VII (Tipping et al., 2011) was used to calculate copper K_D values based on pH, DOC, cations, anions, and the concentration of POC, HFO, HFO, and HMO *at each time step in the simulation*. This method accounts for non-linear partitioning behavior associated with changes in total copper concentration in the water column as well as competition between copper, protons and hardness cations (Ca and Mg) for binding sites. The concentrations of particulate and colloidal HA and FA were calculated from POM and DOC as follows: For both particulate organic matter (POM) and dissolved organic matter (DOM), f_{OC} = 0.58 g org C/g OM (van Hullebusch et al., 2003a; van Hullebusch et al., 2003b); POM is 100% active with 50% HA and 50% FA (Lofts and Tipping, 2000); DOM is 63.5% active with 0% HA and 100% FA (Bryan et al., 2002; Lofts and Tipping, 2011).

Concentrations of HFO and HMO in the water column were estimated from measured total sediment particulate iron and manganese concentrations (Saint Germain les Belles Reservoir), and the water column suspended solids concentrations for the reservoir (Table SI-12). The following specifications were used to determine the amount of HFO and HMO from measured total iron and manganese, respectively.

- Approximately 40% and 18% of the total particulate iron and manganese in the water column were specified to be HFO and HMO, respectively (HydroQual and Manhattan College, 2010)
- HFO-iron was assigned a formula weight of 89 g HFO/mol Fe (Dzombak and Morel, 1990)

- HMO-manganese Mn was assigned a formula weight of 119 g HMO/mol Mn (Tonkin et al., 2004).

The AVS of the sediment in the reservoir was not measured, so it was set at the 50th percentile AVS value of 8.8 µmol/g dry weight from Vangheluwe et al. (2005).

REMOVAL OF COPPER FROM THE WATER COLUMN

Table SI-13: Parameters Used for Summary of Copper Removal from the Water Column

ID	m (mg/L)	v_s (m/d)	S ($g/m^2/d$)	H (m)	log K_D	H/S ($m^3 \cdot d/g$)	t_{30} (days)
Lake Courtille	6.94	0.750	5.21	1.77	4.44	0.340	15.6
St. Germain les Belles	18.0	0.680	12.2	1.60	4.78	0.131	7.06
IME Mesocosms	2.89	0.675	1.95	0.76	5.36	0.390	4.65
MELIMEX	5.91	0.200	1.18	10.0	4.12	8.46	132
Novosibirskoye Reservoir	15.0	0.184	2.76	3.00	5.07	1.09	10.5
TICKET-UWM (Emp Kd)	15.0	2.50	37.5	3.00	4.48	0.0800	4.72
TICKET-UWM (Calc Kd)	15.0	2.50	37.5	3.00	4.90	0.0800	2.75

Averages (from highlighted cells): m = 10.5 mg/L; $K_D = 10^{4.78}$ L/kg

Summary of Schäfers C. 2001. Community level study with copper in aquatic microcosms. Schmallenberg, Germany: Fraunhofer Institute for Molecular Biology and Applied Ecology (IME).

- A study sponsored by the International Copper Association (ICA) represented by European Copper Institute (ECI) was performed at the Fraunhofer Institut for Molecular Biology and Applied Ecology to evaluate effects of a permanent copper sulphate exposure to an aquatic community in indoor microcosms (Schäfers, 2001). Water and sediment were taken from a reference site within the Euro-Ecole project. The study included all important community elements (phyto and zooplankton, macrophytes, benthic invertebrates), which were observed for an exposure period of about 3 months. Copper was tested at permanent nominal concentrations of 5, 10, 20, 40, 80, and 160 μg/L, which were maintained by three treatments weekly.
- The averaged added copper concentrations measured one day after treatment were in the range of 70 – 90 % of the respective nominal concentrations. Within the two or three days between treatments, the dissipation of total copper from the water column was about 25%.

- The exposure period covering spring/early summer up to autumn conditions and the oligotrophic conditions as well as the low hardness and DOC values of the used water can be regarded as a worse case scenario for assessing biological effects.
- Actual primary production of the systems, dominated by macrophytes and indicated by water parameters pH and oxygen content, showed distinctly less increase in spring/early summer at nominal copper concentrations higher than 10 μg/L. At the end of the study, biomass of macrophytes was significantly lower than controls at 40 μg/L (NOEC = 20 μg/L).
- Phytoplankton in total showed higher densities compared to controls at treatments of 20 μg/L and higher (NOEC = 10 μg/L), mainly caused by picoplankton and green algae. Some taxa (Diatomea and Cryptophyta) temporarily showed slightly lower abundances at the same concentrations.
- *Daphnia longispina* and Phyllopoda in general were identified as the most sensitive zooplankton taxa with a NOEC of 20 μg/L. Copepoda and Rotatoria seemed not to be affected or only at the highest treatment level.
- No effects could be detected for benthic macroinvertebrates neither in sediment toxicity tests with microcosm sediments after study termination.
- In total, added copper concentration up to 20 μg/L showed no relevant direct effects on structural endpoints and only slight and temporary effects on functional endpoints represented by oxygen concentrations and pH values. The No Observed Ecologically Adverse Effect Concentration (NOEAEC) of the study can be derived with 20 μg/L, and can be regarded as representative of plankton-dominated waters.

Dissolved Copper Measurements

Dissolved copper measurements were made using anodic stripping voltammetry (ASV) with a Metrohm hanging mercury drop electrode interfaced with a Metrohm 757 Computrace, with a Metrohm Dosimat attached in order to provide accurate automated additions of copper standard. Table SI-14 below contains the dissolved copper results used in present study.

Table SI-14. Dissolved Copper Measurements

Microcosm	Time after 1st treatment	Dissolved Copper, µg/L
Control (pooled)	1h	1.7
Control (pooled)	24h	2.3
Control (pooled)	48h	2.7
No. 25 (5 µg/L)	1h	6.2
No. 25 (5 µg/L)	24h	6.4
No. 25 (5 µg/L)	48h	5.5
No. 30 (5 µg/L)	1h	8.8
No. 30 (5 µg/L)	24h	6.7
No. 30 (5 µg/L)	48h	6.2
No. 21 (10 µg/L)	1h	16.7
No. 21 (10 µg/L)	24h	9.4
No. 21 (10 µg/L)	48h	10
No. 33 (10 µg/L)	1h	15.6
No. 33 (10 µg/L)	24h	9.8
No. 33 (10 µg/L)	48h	9
No. 27 (20 µg/L)	1h	21.3
No. 27 (20 µg/L)	24h	14.5
No. 27 (20 µg/L)	48h	13.9
No. 34 (20 µg/L)	1h	15.2
No. 34 (20 µg/L)	24h	17.4
No. 34 (20 µg/L)	48h	-
No. 24 (40 µg/L)	1h	38.4
No. 24 (40 µg/L)	24h	32.4
No. 24 (40 µg/L)	48h	25.1
No. 36 (40 µg/L)	1h	41.5
No. 36 (40 µg/L)	24h	33.8
No. 36 (40 µg/L)	48h	31.3
No. 26 (80 µg/L)	1h	67.2
No. 26 (80 µg/L)	24h	41.2
No. 26 (80 µg/L)	48h	41
No. 35 (80 µg/L)	1h	58.6
No. 35 (80 µg/L)	24h	40.6
No. 35 (80 µg/L)	48h	48.6
No. 23 (160 µg/L)	1h	151.9
No. 23 (160 µg/L)	24h	63.8
No. 23 (160 µg/L)	48h	57.7
No. 31 (160 µg/L)	1h	142
No. 31 (160 µg/L)	24h	70.9
No. 31 (160 µg/L)	48h	55.6

Total Copper Measurements

Total copper measurements were made following the international standard method ISO 11885 / DEV E22. A Liberty II ICP-OES from Varian (Eschborn) was used. Table SI-15 below contains the copper results used in present study.

Table SI-15 Total Copper Measurements

**Microcosm**	**Total Copper, µg/L**
B32 control 1	*3.4*
B29 control 2	*3.9*
B22 control 3	*5.3*
B28 control 4	**10**
B25 5 µg/L	**11.9**
B30 5 µg/L	**14.2**
B21 10 µg/L	**19.7**
B33 10 µg/L	**21.7**
B27 20 µg/L	**22.7**
B34 20 µg/L	**36.1**
B24 40 µg/L	**40**
B36 40 µg/L	**54.1**
B26 80 µg/L	**58.3**
B35 80 µg/L	**65.7**
B23 160 µg/L	**114**
B31 160 µg/L	**130**
TM28 (6.3 µg/L Cu)	*6.3*
Limit of determination	**8.9**

Italics: data below limit of determination (only for information; bold: valid data within working range.
Sampling date is 2/22/2001

REFERENCES

AMINAL/AWZ, 1995. Methodological study on the inventory, the ecological effects and remediation of sediments of Flemish watercourses (in Dutch), Final report, p. 209.

ARCHE, 2013. Unit World Model - Overview of available data on relevant environmental parameters, Gent, Belgium.

Besser, J.M., Brumbaugh, W.G., Kemble, N.E., Ivey, C.D., Kunz, J.L., Ingersoll, C.G., Rudel, D., 2011. Toxicity of nickel-spiked freshwater sediments to benthic invertebrates—Spiking methodology, species sensitivity, and nickel bioavailability. U.S. Geological Survey, Reston, Virginia.

Bryan, S.E., Tipping, E., Hamilton-Taylor, J., 2002. Comparison of measured and modelled copper binding by natural organic matter in freshwaters. Comparative Biochemistry and Physiology Part C: Toxicology & Pharmacology 133, 37-49.

Canavan, R.W., Van Cappellen, P., Zwolsman, J.J.G., van den Berg, G.A., Slomp, C.P., 2007. Geochemistry of trace metals in a fresh water sediment: Field results and diagenetic modeling. Science of The Total Environment 381, 263-279.

Chapra, S.C., 1997. Surface Water-Quality Modeling. McGraw-Hill, New York.

de Deckere, E., De Cooman, W., Florus, M., Meire, P., 2000. Characterization of sediments in Flanders. Monitoring reports navigable watercourses 1999-2000., UIA, Biology Department/AMINAL division water.

Di Toro, D.M., Allen, H.E., Bergman, H.L., Meyer, J.S., Paquin, P.R., Santore, R.C., 2001. Biotic ligand model of the acute toxicity of metals. 1. Technical Basis. Environ. Toxicol. Chem. 20, 2383-2396.

Di Toro, D.M., McGrath, J.A., Hansen, D.J., Berry, W.J., Paquin, P.R., Mathew, R., Wu, K.B., Santore, R.C., 2005. Predicting sediment metal toxicity using a sediment biotic ligand model: Methodology and initial application. Environ. Toxicol. Chem. 24, 2410-2427.

Di Toro, D.M., O'Connor, D.J., Thomann, R.V., St. John, J.P., 1981. Analysis of Fate of Chemicals in Receiving Waters, Phase 1. Chemical Manufacturers Association, Washington, DC. Prepared by HydroQual, Inc., Mahwah, NJ.

Diamond, M.L., Mackay, D., Cornett, R.J., Chant, L.A., 1990. A model of the exchange of inorganic chemicals between water and sediments. Environ. Sci. Technol. 24, 713-722.

Dyrssen, D., Kremling, K., 1990. Increasing hydrogen sulfide concentration and trace metal behavior in the anoxic Baltic waters. Marine Chemistry 30, 193-204.

Dzombak, D.A., Morel, F.M.M., 1990. Surface complexation modeling : hydrous ferric oxide. Wiley, New York.

EC, 2004. European Union System for the Evaluation of Substances 2.0 (EUSES). Prepared for the European Chemicals Bureau by the National Institute of Public Health and the Environment (RIVM), Bilthoven, The Netherlands.

EURAS, 2008. Acid Volatile Sulfide survey of large river sediments in the United Kingdom, Spain, and Finland in 2007 - Working Draft. Prepared for the Ecotoxicity Technical Advisory Panel.

European Chemicals Agency, 2010. Chapter R.16: Environmental Exposure Estimation, Guidance on Information Requirements and Chemical Safety Assessment. European Chemicals Agency, Helsinki, Finland.

Farley, K.J., Carbonaro, R.F., Di Toro, D.M., 2007. Unit World Model Tier 1 Hazard Ranking Model for Metals in Lakes. Manhattan College, Riverdale, New York.

Farley, K.J., Carbonaro, R.F., Fanelli, C.J., Costanzo, R., Rader, K.J., Di Toro, D.M., 2011. TICKET-UWM: A coupled kinetic, equilibrium, and transport screening model for metals in lakes. Environ. Toxicol. Chem. 30, 1278-1287.

Farley, K.J., Rader, K.J., Miller, B.E., 2008. Tableau Input Coupled Kinetic Equilibrium Transport (TICKET) Model. Environ. Sci. Technol. 42, 838-844.

Gallon, C., Tessier, A., Gobeil, C., Alfaro-De La Torre, M.C., 2004. Modeling diagenesis of lead in sediments of a Canadian Shield lake. Geochim. Cosmochim. Acta 68, 3531-3545.
Gee, A.K., Bruland, K.W., 2002. Tracing Ni, Cu, and Zn kinetics and equilibrium partitioning between dissolved and particulate phases in South San Francisco Bay, California, using stable isotopes and high-resolution inductively coupled plasma mass spectrometry. Geochim. Cosmochim. Acta 66, 3063-3083.
Heijerick, D., Van Sprang, P., 2005. Appendix ENV F - Review of copper partitioning coefficients in the aquatic environment and processes causing the observed variation., Copper, Copper II Sulfphate, Pentahydrate, Copper(I)Oxide, Copper(Ii)Oxide, Dicopper Chloride Trihydroxide, pp. F1 - F40.
Herzl, V.M.C., Millward, G.E., Wollast, R., Achterberg, E.P., 2003. Species of dissolved Cu and Ni and their adsorption kinetics in turbid riverwater. Estuarine, Coastal and Shelf Science 56, 43-52.
Hunt, C.D., Smith, D.L., 1983. Remobilization of Metals from Polluted Marine Sediments. Can. J. Fish. Aquat. Sci. 40, s132-s142.
HydroQual, Manhattan College, 2010. Development of Models for Metal Partitioning to Oxic Sediments.
Klinkhammer, G., Heggie, D.T., Graham, D.W., 1982. Metal diagenesis in oxic marine sediments. Earth and Planetary Science Letters 61, 211-219.
Lofts, S., Tipping, E., 2000. Solid-solution metal partitioning in the Humber rivers: application of WHAM and SCAMP. Science of The Total Environment 251, 381-399.
Lofts, S., Tipping, E., 2011. Assessing WHAM/Model VII against field measurements of free metal ion concentrations: model performance and the role of uncertainty in parameters and inputs. Environmental Chemistry 8, 501-516.
Mackay, D., 1979. Finding fugacity feasible. Environ. Sci. Technol. 13, 1218-1223.
Mackay, D., 1991. Multimedia environmental models : the fugacity approach. Lewis Publishers, Chelsea, Michigan.
Mackay, D., Paterson, S., 1991. Evaluating the multimedia fate of organic chemicals: a level III fugacity model. Environ. Sci. Technol. 25, 427-436.
Millward, G.E., Glegg, G.A., Morris, A.W., 1992. Zn and Cu removal kinetics in estuarine waters. Estuarine, Coastal and Shelf Science 35, 37-54.
Santore, R.C., Di Toro, D.M., Paquin, P.R., Allen, H.E., Meyer, J.S., 2001. Biotic ligand model of the acute toxicity of metals. 2. Application to acute copper toxicity in freshwater fish and Daphnia. Environ. Toxicol. Chem. 20, 2397-2402.
Schäfers, C., 2001. Community level study with copper in aquatic microcosms. Fraunhofer Institute for Molecular Biology and Applied Ecology (IME), Schmallenberg, Germany, p. 109.
Schecher, W.D., McAvoy, D.C., 2003. MINEQL+: A Chemical Equilibrium Modeling System, Version 4.5 for Windows, User's Manual, Second ed. Environmental Research Software, Hallowell, Maine.
Shaw, T.J., Gieskes, J.M., Jahnke, R.A., 1990. Early diagenesis in differing depositional environments: The response of transition metals in pore water. Geochim. Cosmochim. Acta 54, 1233-1246.
Simpson, S.L., Rosner, J., Ellis, J., 2000. Competitive displacement reactions of cadmium, copper, and zinc added to a polluted, sulfidic estuarine sediment. Environ. Toxicol. Chem. 19, 1992-1999.
Skeaff, J.M., Dubreuil, A.A., Brigham, S.I., 2002. The concept of persistence as applied to metals for aquatic hazard identification. Environ. Toxicol. Chem. 21, 2581-2590.
Stumm, W., Morgan, J.J., 1996. Aquatic chemistry : chemical equilibria and rates in natural waters. Wiley, New York.
Tipping, E., 1993. Modelling ion binding by humic acids. Colloids and Surfaces A: Physicochemical and Engineering Aspects 73, 117-131.
Tipping, E., 1994. WHAM--a chemical equilibrium model and computer code for waters, sediments, and soils incorporating a discrete site/electrostatic model of ion-binding by humic substances. Comput. Geosci. 20, 973-1023.
Tipping, E., 1998. Humic Ion-Binding Model VI: An Improved Description of the Interactions of Protons and Metal Ions with Humic Substances. Aquat. Geochem. 4, 3-47.

Tipping, E., Hurley, M.A., 1992. A unifying model of cation binding by humic substances. Geochim. Cosmochim. Acta 56, 3627-3641.
Tipping, E., Lofts, S., Sonke, J.E., 2011. Humic Ion-Binding Model VII: a revised parameterisation of cation-binding by humic substances. Environmental Chemistry 8, 225-235.
Tonkin, J.W., Balistrieri, L.S., Murray, J.W., 2004. Modeling sorption of divalent metal cations on hydrous manganese oxide using the diffuse double layer model. Appl. Geochem. 19, 29-53.
United Nations, 2017. Globally Harmonized System of Classification and Labelling of Chemicals (GHS), Seventh ed. United Nations, New York and Geneva.
van den Berg, G.A., Loch, J.P.G., van der Heijdt, L.M., Zwolsman, J.J.G., 1999. Mobilisation of heavy metals in contaminated sediments in the river Meuse, The Netherlands. Water Air Soil Pollut. 116, 567-586.
van Hullebusch, E., Chatenet, P., Deluchat, V., Chazal, P.M., Froissard, D., Botineau, M., Ghestem, A., Baudu, M., 2003a. Copper accumulation in a reservoir ecosystem following copper sulfate treatment (St. Germain Les Belles, France). Water Air Soil Pollut. 150, 3-22.
van Hullebusch, E., Chatenet, P., Deluchat, V., Chazal, P.M., Froissard, D., Lens, P.N.L., Baudu, M., 2003b. Fate and forms of Cu in a reservoir ecosystem following copper sulfate treatment (Saint Germain les Belles, France). Journal de Physique IV 107, 1333-1336.
Vangheluwe, M., Heijerick, D., Verdonck, F., 2005. Probabilistic assessment of copper bioavailability in sediments. Prepared for the International Copper Association performed by EURAS, Belgium.
Wersin, P., Höhener, P., Giovanoli, R., Stumm, W., 1991. Early diagenetic influences on iron transformations in a freshwater lake sediment. Chemical Geology 90, 233-252.
Xue, H.B., Gachter, R., Sigg, L., 1997. Comparison of Cu and Zn cycling in eutrophic lakes with oxic and anoxic hypolimnion. Aquat. Sci. 59, 176-189.
Zwolsman, J.J.G., VanEck, B.T.M., VanderWeijden, C.H., 1997. Geochemistry of dissolved trace metals (cadmium, copper, zinc) in the Scheldt estuary, southwestern Netherlands: Impact of seasonal variability. Geochim. Cosmochim. Acta 61, 1635-1652.
